# The benzylisoquinoline alkaloids, berberine and coptisine, act against camptothecin-resistant topoisomerase I mutants

**DOI:** 10.1038/s41598-021-87344-2

**Published:** 2021-04-08

**Authors:** Naomi Inoue, Takeshi Terabayashi, Yuri Takiguchi-Kawashima, Daisuke Fujinami, Shigeru Matsuoka, Masanori Kawano, Kazuhiro Tanaka, Hiroshi Tsumura, Toshimasa Ishizaki, Hisashi Narahara, Daisuke Kohda, Yoshihiro Nishida, Katsuhiro Hanada

**Affiliations:** 1 Department of Obstetrics and Gynecology, Faculty of Medicine, Oita University, 1-1 Idaigaoka, Hasama-machi, Yufu, Oita 879-5593 Japan; 2 Department of Pharmacology, Faculty of Medicine, Oita University, Yufu, Japan; 3 Clinical Engineering Research Center, Faculty of Medicine, Oita University, 1-1 Idaigaoka, Hasama-machi, Yufu, Oita 879-5593 Japan; 4 Division of Structural Biology, Medical Institute of Bioregulation, Kyushu University, Fukuoka, Japan; 5 Department of Clinical Biology Ant Therapeutics, Faculty of Medicine, Oita University, Yufu, Japan; 6 Department of Orthopaedic Surgery, Faculty of Medicine, Oita University, Yufu, Japan; 7 Department of Life Sciences, Graduate School of Arts and Sciences, The University of Tokyo, Tokyo, Japan

**Keywords:** Biochemistry, Biotechnology, Cancer, Chemical biology, Drug discovery, Genetics, Molecular biology

## Abstract

DNA replication inhibitors are utilized extensively in studies of molecular biology and as chemotherapy agents in clinical settings. The inhibition of DNA replication often triggers double-stranded DNA breaks (DSBs) at stalled DNA replication sites, resulting in cytotoxicity. In East Asia, some traditional medicines are administered as anticancer drugs, although the mechanisms underlying their pharmacological effects are not entirely understood. In this study, we screened Japanese herbal medicines and identified two benzylisoquinoline alkaloids (BIAs), berberine and coptisine. These alkaloids mildly induced DSBs, and this effect was dependent on the function of topoisomerase I (Topo I) and MUS81-EME1 structure-specific endonuclease. Biochemical analysis revealed that the action of BIAs involves inhibiting the catalytic activity of Topo I rather than inducing the accumulation of the Topo I-DNA complex, which is different from the action of camptothecin (CPT). Furthermore, the results showed that BIAs can act as inhibitors of Topo I, even against CPT-resistant mutants, and that the action of these BIAs was independent of CPT. These results suggest that using a combination of BIAs and CPT might increase their efficiency in eliminating cancer cells.

## Introduction

Natural medicines are commonly used worldwide for the treatment of endocrine and metabolic diseases, infections, and inflammatory symptoms^1^. In Japan, these medications are often combined with Western medicines to induce synergistic effects or mitigate the side effects of Western medicines^1–3^. Few natural medicines for cancer treatment are well understood^4^. However, numerous prototype compounds utilized as anticancer agents were discovered from medicinal plants, including camptothecin (CPT), etoposide, taxanes, and vinca alkaloids, and from bacteria, such as mitomycin and anthracyclines. Although these agents act against cancer using a variety of mechanisms, they can be classified into two major groups, namely, DNA replication inhibitors and mitosis inhibitors. DNA replication inhibitors are most frequently used in cancer treatment. CPT inhibits Topo I activity, and etoposide and anthracyclines inhibit topoisomerase II (Topo II) activity^5^. Because topoisomerases play a crucial role in DNA replication and chromosome partitioning, the inhibition of their activities results in the formation of DSBs in proliferating cells, which leads to selective cell death^5,6^. Mitomycin C tethers both strands of the DNA to yield interstrand DNA crosslinks, which results in the inhibition of DNA replication and DSB formation^7,8^. Mitosis inhibitors have also been discovered in medicinal plants. Taxanes stabilize the microtubule polymer and prevent its disassembly^9^, and vinca alkaloids block the polymerization of β-tubulin^10^. Therefore, treatments using taxanes and vinca alkaloids induce defects in chromosomal segregation^9,10^.

Among the natural compounds used as anticancer agents, many researchers have actively investigated the mechanism underlying the resistance to CPT. CPT is classified as a monoterpene indole alkaloid and is produced by plants, such as *Camptotheca acuminata*, *Ophiorrhiza pumila*, *Ophiorrhiza liukiuensis*, and *Nothapodytes foetida*^11,12^. CPT functions to stabilize the DNA-Topo I complex, which results in the formation of single-strand breaks (SSBs) on the DNA^13^. The SSB formed by the Topo I-DNA complex might then be converted to a DSB through progression of the DNA replication fork^14^. The catalytic activity of Topo I is to cleave one of the two strands of double-stranded DNA and relax supercoiled DNA. The OH group of Tyr723 in human Topo I is the active site and cuts the phosphate group at the 3′-end of the strand to form a binary DNA-Topo I covalent complex. After cleavage, the broken DNA strand can rotate around the unbroken strand and remove DNA supercoils, and cleaved strand is then rejoined. Thus, Topo I controls the topological state of DNA. Supercoiled DNAs typically appear in front of replication forks during DNA replication, and the dysfunction of Topo I due to treatment causes stalled replication forks^15^. Thus, CPT induces the accumulation of not only DNA replication-mediated DSB formation^14^ but also stalls DNA replication forks^15^, which results in cytotoxicity in proliferating cells. CPT is toxic to most eukaryotic organisms, such as yeasts, insects, plants, and mammals. Clearly, CPT-producing plants possess defence mechanisms to protect themselves against CPT. Topo I in CPT-producing plants contains specific amino acid substitutions that affect its resistance to CPT, such as N421K, L530I, and/or N722S (numbered according to human Topo I^11^). Because these residues are crucial for the interaction between CPT and Topo I, such substitutions lead to defects in this interaction and result in resistance in CPT-producing plants^11^. Similarly, CPT-resistant mutations in the *TOP1* gene have also been discovered in human cancer cell lines that were generated as CPT-resistant lines in vitro, namely, R346H and D533G^16^. These residues also play an essential role in the interaction between CPT and Topo I. To date, many other mutations responsible for CPT resistance have been identified in the *TOP1* gene^17–19^. Although we only mentioned CPT, cytotoxic substances produced by organisms are usually coupled to defence mechanisms to avoid self-toxicity.

For overcoming drug resistance in cancer cells, it is crucial to identify a compound that acts against drug-resistant cells via a mechanism that differs from that of existing agents and to combine the identified compound with known agents in chemotherapy regimens^16,20^. Therefore, further screening to discover a variety of substances that inhibit both DNA replication and mitosis via novel mechanisms of action is worthwhile. In this study, we screened herbal extracts used as ingredients in Japanese natural medicines to identify compounds that induce DSBs and, more specifically, to identify DSB formation at DNA replication sites. The screening revealed two candidates, and further characterization of these substances, namely, berberine and coptisine, found that these compounds might act against cancer cells with CPT-resistant *TOP1* mutations.

## Results

### Screening of potential anticancer compounds from Japanese herbal medicines

**Figure 1:**
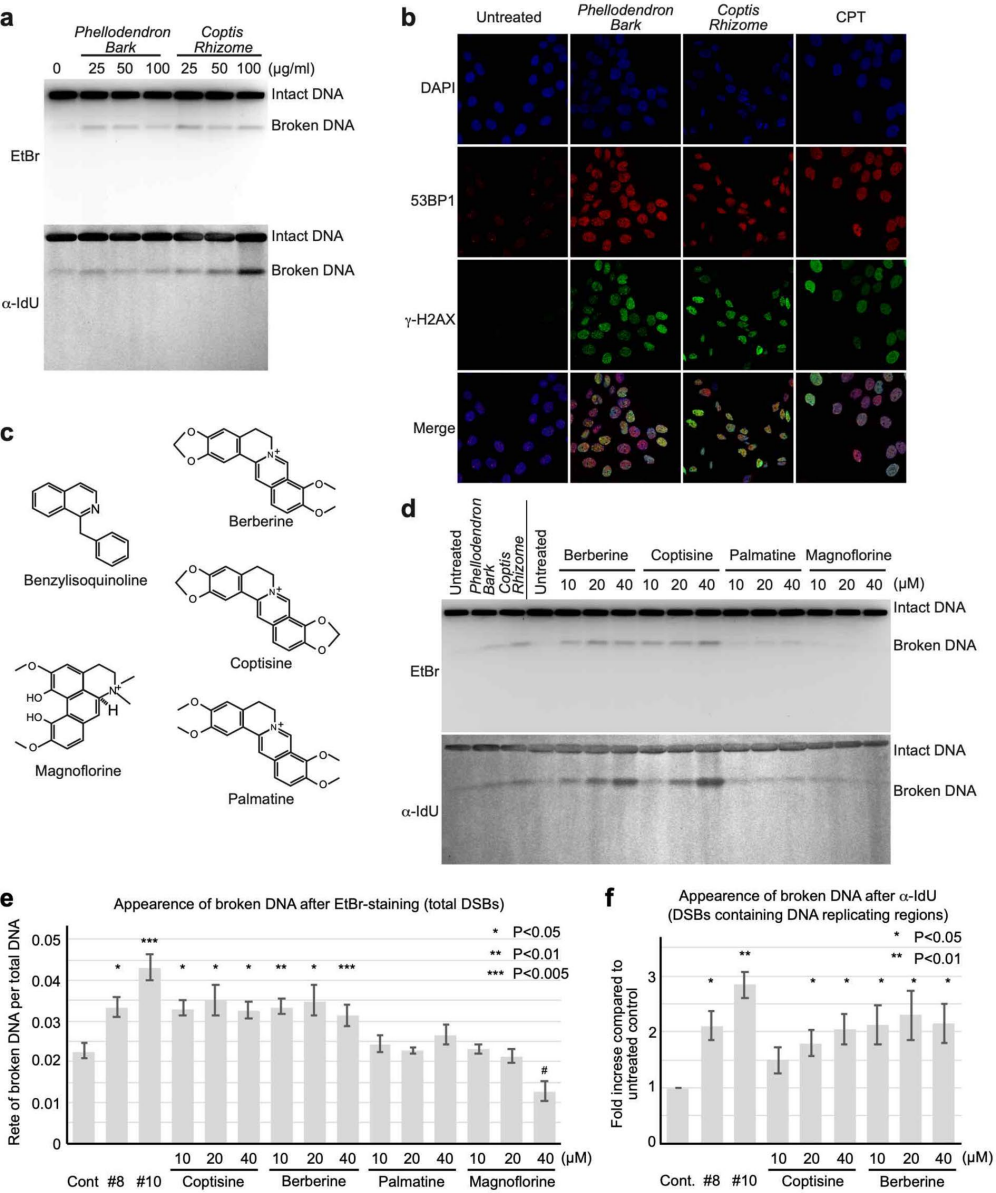
Screening of herbal extracts and characterization of berberine and coptisine. (**a**) PFGE analysis of DSB accumulation after treatment with extracts of *Phellodendron Bark* and *Coptis Rhizome*. Total broken DNA was detected by ethidium bromide (EtBr) staining, whereas DSBs at DNA replication sites were detected by immunoblotting with the anti-BrdU antibody. (**b**) Immunofluorescence analysis of DSB accumulation by detecting the DSB markers γ-H2AX and 53BP1 foci. (**c**) Molecular structures of the BIAs berberine, coptisine, palmatine, and magnoflorine. (**d**) PFGE analysis of DSB accumulation after treatment with berberine, coptisine, palmatine, and magnoflorine. (**e**) Quantification of broken DNA. The data are presented as the ratios of the amount of broken DNA per total DNA (intact DNA + broken DNA). (**f**) Quantification of broken DNA. The data are presented as fold inductions relative to the untreated control.

To discover novel anticancer substances, herbal extracts used in Japanese natural medicines were screened by pulse field gel electrophoresis (PFGE)^6^. We analysed the appearance of the DNA fragments from 500 kbp to several Mbp as ‘broken DNA’ (the size marker is shown in Supplementary Fig. 1b). Under this condition, the DNA fragments from 500 kbp to several Mbp were compacted into one band in the PFGE gel. Detailed protocols^6,21^ and the sensitivity of this assay^22^ were previously reported^6,21,22^. We believe that many cancer cell lines have their own characteristics. Even the sensitivities of colon cancer cell lines to a particular drug can be quite different. Therefore, we used a neutral cell line rather than a particular type of cancer cell line in our screening. Treatment with two of the 120 herbal extracts (Supplementary Table 1), namely two extracts prepared from Phellodendron bark (#8) and Coptis rhizome (#10), induced accumulation of DSBs (Supplementary Fig. 1). These findings were confirmed by PFGE (Fig. 1a, Supplementary Fig. 2a). The accumulation of DSBs induced by the treatments with herbal extracts of Phellodendron bark and Coptis rhizome was also confirmed through fluorescence microscopy by visualizing the DSB marker antigens 53BP1 and γ-H2AX (Fig. 1b). We therefore analysed the open data in the Traditional Medicine & Pharmaceutical Database (TradMPD) published by the Institute of Natural Medicine of Toyama University (http://dentomed.toyama-wakan.net/en/chemical_analysis_result_of_crude_drug_extracts/). Typical nutrients, such as sugars, including amino saccharides and saccharic acids, fat, including fatty acids and steroids, amino acids, vitamins, and minerals, were omitted. Although these medicines are prepared from unrelated ingredients (see Supplementary Table 1), both contain structurally similar BIAs as their major components, namely, berberine, coptisine, and palmatine^23^ (Fig. 1c). With the aim of understanding the chemical characteristics, these BIAs were analysed to determine whether they were responsible for the formation of DSBs. Cells treated with berberine and coptisine, but not palmatine, showed significant accumulation of DSBs (Fig. 1b,d,e). Because broken DNA contained 5-iodo-2′-deoxyuridine (IdU), BIA-induced DSBs occurred around DNA replication sites (Fig. 1d,f). Another type of BIA, magnoflorine, was used as a negative control, and the analysis showed that magnoflorine did not induce DSB formation (Fig. 1d,e, Supplementary Fig. 2b).

**Figure 2:**
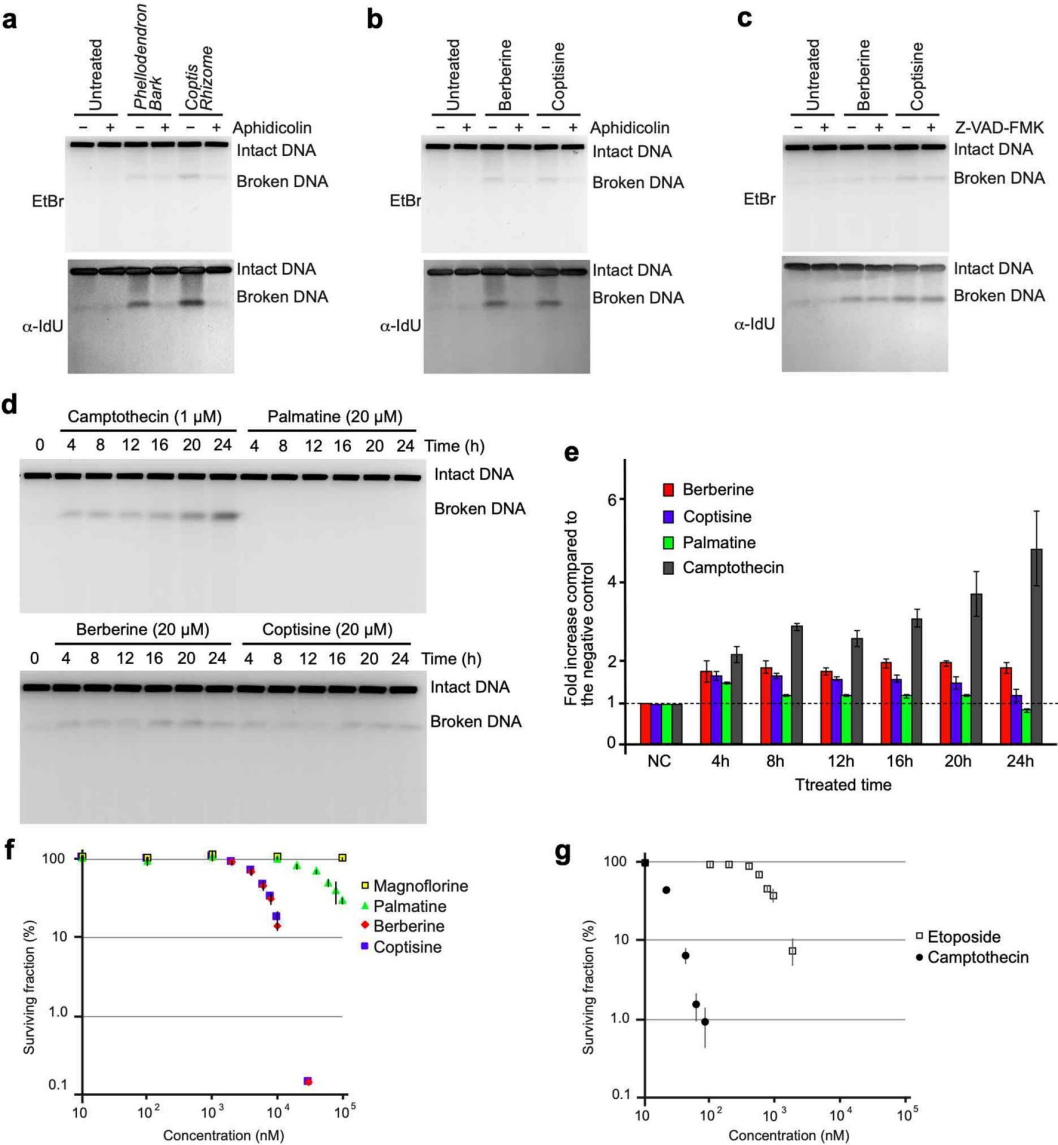
Analysis of the cytotoxic effects of berberine, coptisine, and palmatine. (**a**) PFGE analysis of DSB accumulation after treatment with aphidicolin combined with extracts of *Phellodendron Bark* and *Coptis Rhizome*. (**b**) PFGE analysis of DSB accumulation after treatment with aphidicolin combined with extracts of berberine and coptisine. (**c**) PFGE analysis of DSB accumulation after treatment with Z-VAD-FMK combined with extracts of berberine and coptisine. (**d**) Time-course PFGE analysis of DSB accumulation after treatment with CPT, palmatine, berberine and coptisine. (**e**) Quantification of broken DNA. The data are presented as fold inductions relative to the untreated control. (**f**) Survival curves of MRC5sv cells against berberine, coptisine, palmatine, and magnoflorine. (**g**) Survival curves of MRC5sv cells treated with CPT and etoposide.

### Characterization of BIA-induced DSB formation

Previous studies have shown that DSBs induced by topoisomerase inhibitors do not accumulate after combined treatment with aphidicolin^14,24^ (Supplementary Fig. 3); thus, the progression of DNA replication forks appears to be associated with topoisomerase-dependent DSB formation. Therefore, we assessed whether combined treatment with aphidicolin could suppress the formation of BIA-induced DSBs. DSBs induced by herbal extracts from Phellodendron bark and Coptis rhizome were suppressed by combined treatment with aphidicolin (Fig. 2a, Supplementary Fig. 4a). Subsequently, the DSBs induced by treatment with berberine and coptisine were examined. Both the induction of DSBs by berberine and coptisine was suppressed by combined treatment with aphidicolin (Fig. 2b, Supplementary Fig. 4b). The DSBs suppressed by combined treatment with aphidicolin contained IdU, which suggests that BIA-induced DSBs primarily occurred at DNA replication sites. To assess whether BIA-induced DSBs were produced as a result of apoptosis, we examined whether BIA-induced DSB formation occurred in the presence of the apoptotic nuclease inhibitor Z-VAD-FMK^25^. As expected, BIA-induced DSB formation was not suppressed by combined treatment with Z-VAD-FMK (Fig. 2c, Supplementary Fig. 4c). These results indicate that the DSBs induced by the aforementioned alkaloids are associated with DNA replication rather than apoptosis. We then followed the time course of BIA-induced DSBs in comparison to those induced by CPT. During treatment with CPT, DSBs started to accumulate after 4 h, and this accumulation continued to increase until 24 h (Fig. 2d,e). However, only a slight accumulation of DSBs was observed after 4 h of treatment with berberine and coptisine, but no clear increase was observed from 4 to 24 h. This finding was different from the CPT results. An accumulation of DSBs was hardly observed after treatment with palmatine (Fig. 2d,e, Supplementary Fig. 5). To test its cytotoxic effect, we generated dose-dependent cell survival curves (Fig. 2f,g). MRC5sv cells were treated with the indicated compounds for 24 h, and the medium was then refreshed. Following incubation, survival curves were determined based on the percentages of colony formation. The survival curves suggested that berberine and coptisine showed higher toxicities than palmatine; the lethal dose 50 (LD_50_) for berberine and coptisine was 8 μM, whereas that for palmatine was 80 μM. In addition, magnoflorine showed no toxicity until 100 μM (Fig. 2f). These results are consistent with those from previous studies that assessed the cytotoxic activities of berberine and coptisine^26,27^. However, the cytotoxicities of berberine and coptisine was milder than those of etoposide and CPT, which are used in chemotherapy in the clinic (Fig. 2f,g).

**Figure 3:**
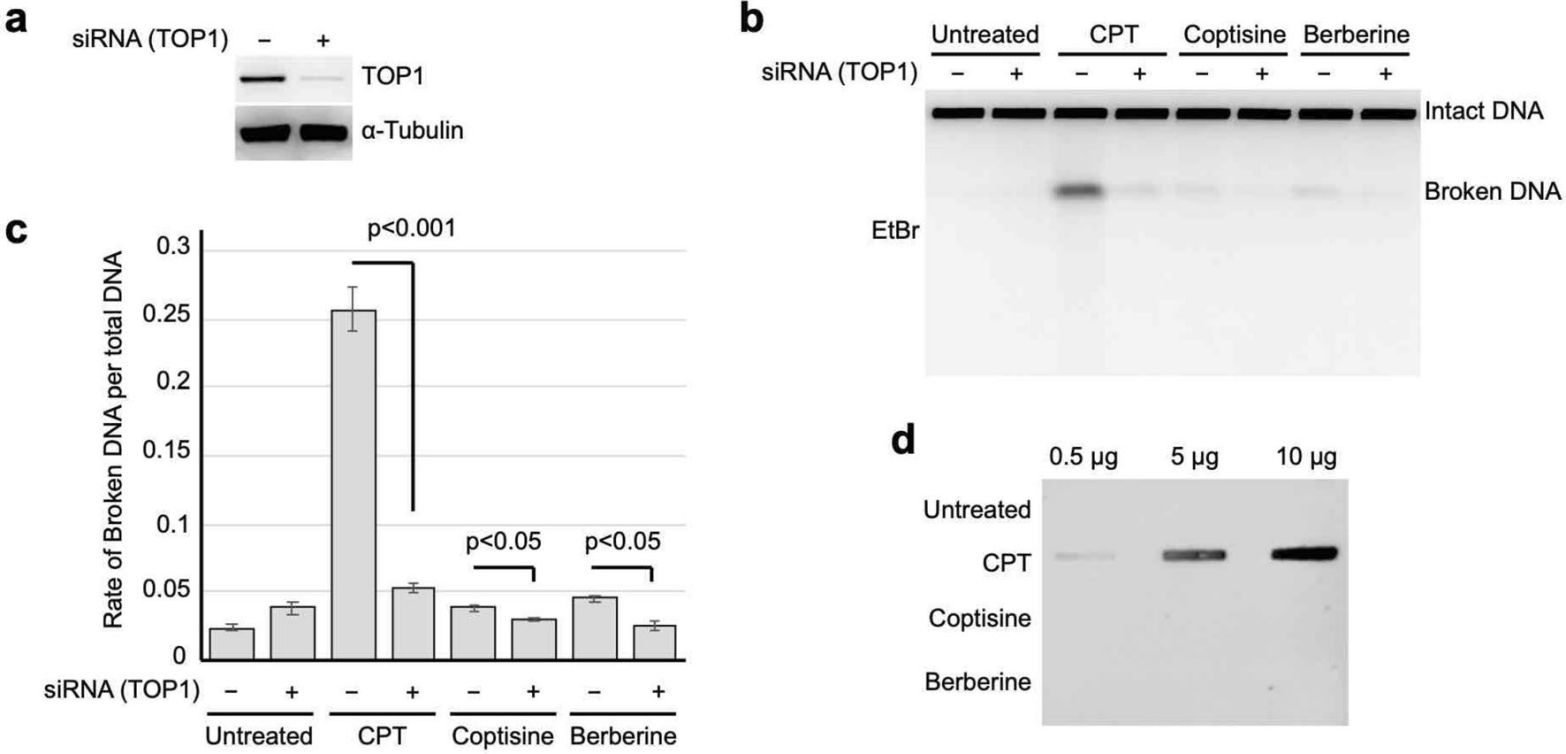
Analysis of Topo I inhibition by BIAs in vivo. (**a**) Western blot analysis of Topo I after transfection with siRNA against the *Top1* gene. (**b**) PFGE analysis of DSB accumulation after treatment with transient Topo I depletion by siRNA combined with berberine and coptisine. (**c**) Quantification of broken DNA per total DNA after treatment with transient Topo I depletion by siRNA combined with berberine and coptisine. The means and SEs were determined from four independent experiments. (**d**) Analysis of the accumulation of the Topo I-DNA complex by ICE assay. Following the manufacturer’s recommended protocol, the cells were treated with 50 μM CPT, coptisine, and berberine, and the Topo I-DNA complexes were visualized by immunoblotting using an anti-Topo I antibody.

**Figure 4:**
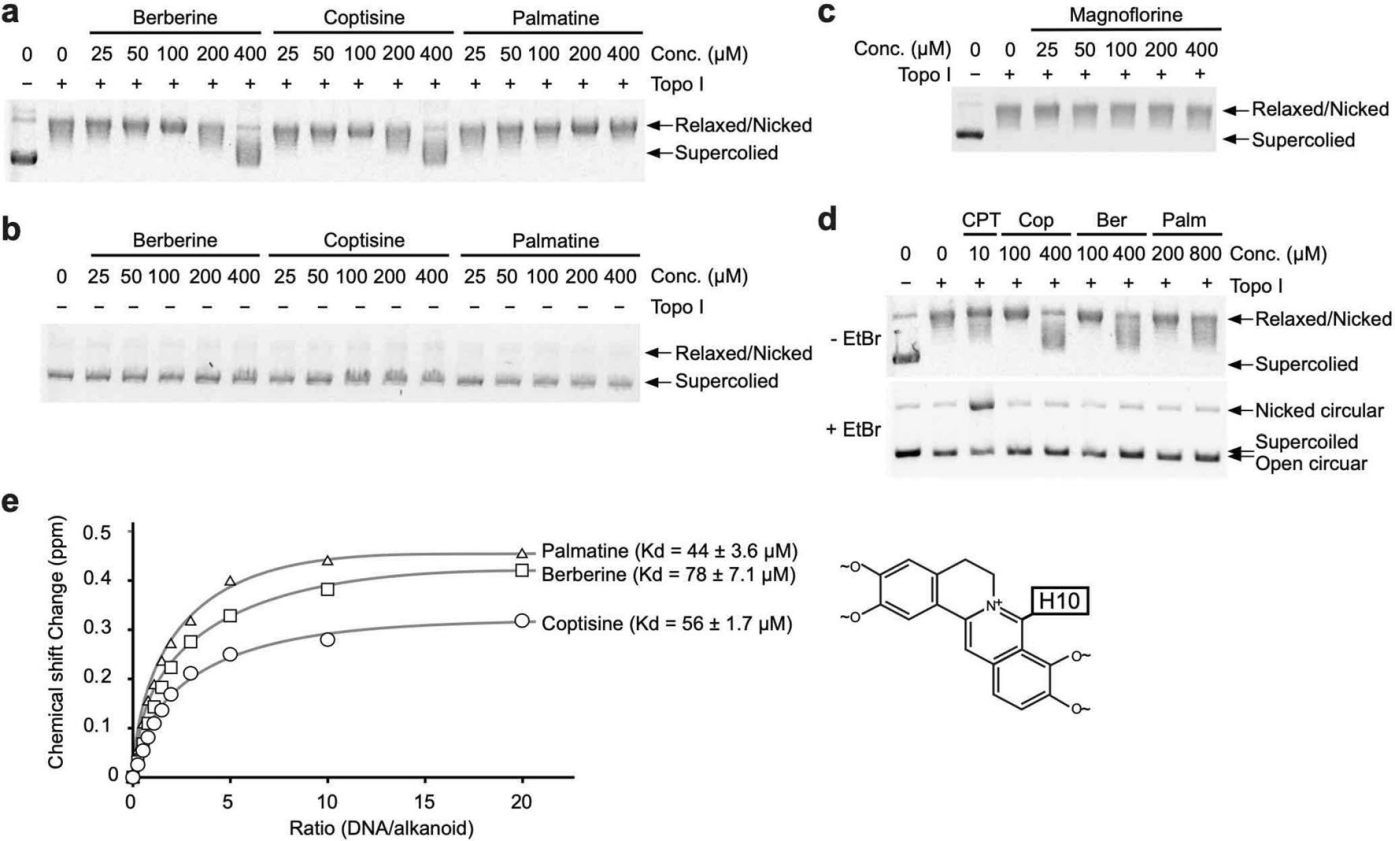
Analysis of Topo I inhibition by BIAs in vitro. (**a,b**) Inhibition of the relaxation activity of Topo I by berberine, coptisine, and palmatine. (**c**) Relaxation activity of Topo I after treatment with magnoflorine. (**d**) Detection of nicked DNA based on the effects on the relaxation activity of Topo I by CPT, coptisine (Cop), berberine (Ber), and palmatine (Palm). (**d**) NMR titration curves, ^1^H chemical shift changes versus the concentration of dsDNA, and dissociation constant (Kd) values for coptisine, berberine, and palmatine. The inset shows the proton in the alkaloid backbone used for the NMR analysis.

**Figure 5:**
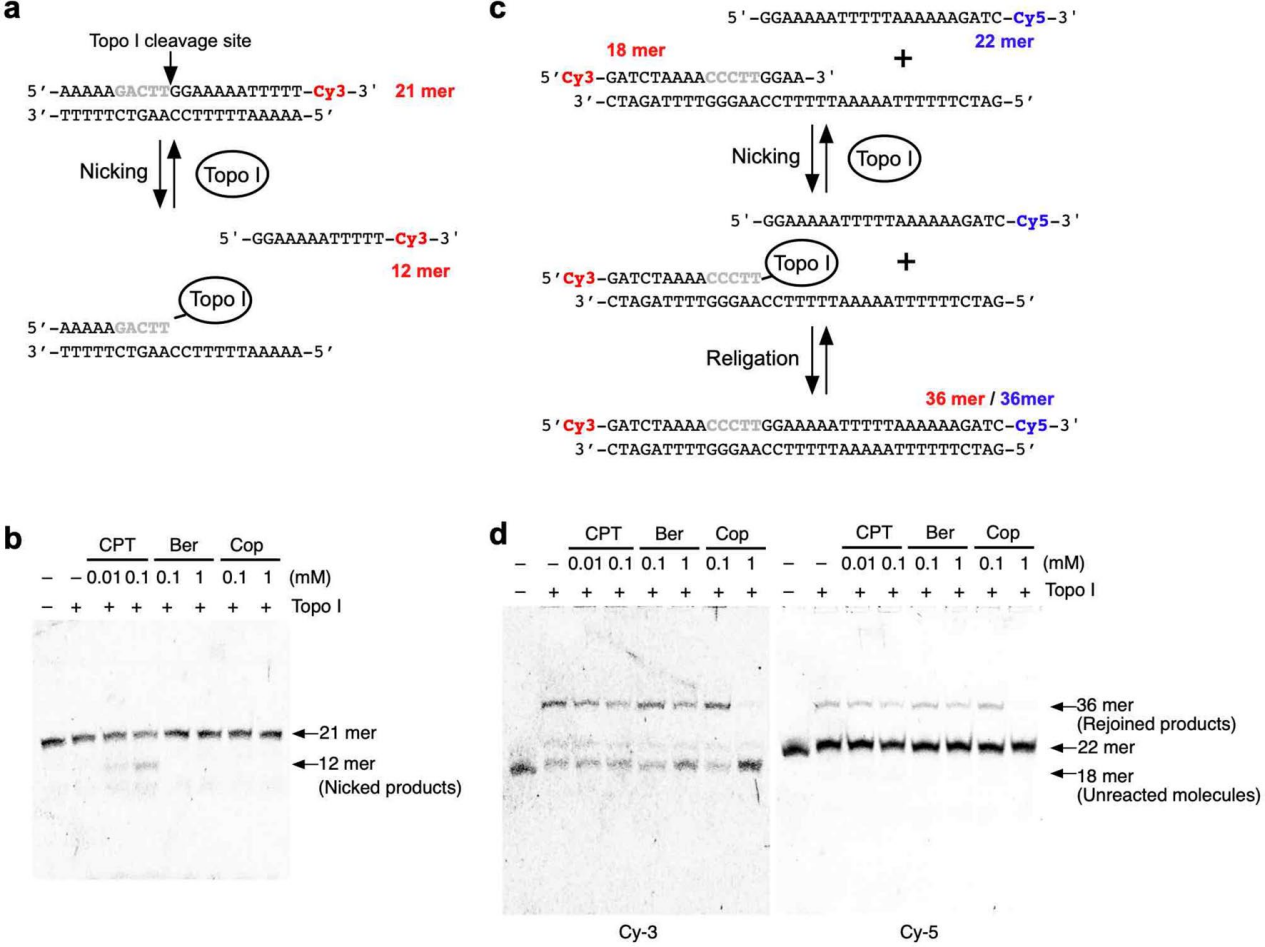
Analysis of the inhibitory effects of BIAs in nicking and rejoining assays. (**a**) Schematic representation of the nicking assay. Nicked DNA products were detected by the Cy-3 fluorescent signal. Unaffected DNA molecules were detected as 36-mer fragments, and nicked DNAs were detected as 22-mer fragments. (**b**) Inhibitory effects of treatment with CPT, berberine (Ber), and coptisine (Cop) on Topo I-dependent nicking activity. (**c**) Schematic representation of the rejoining assay. The rejoined products were detected by Cy-3 and Cy-5 fluorescent signals. Unaffected DNA molecules were detected as 18-mer fragments on a Cy-3-visualized gel, and nicked DNAs were detected as 36-mer fragments on both the Cy-3- and Cy-5-visualized gels. (**d**) Inhibitory effects of treatment with CPT, berberine (Ber), and coptisine (Cop) on Topo I-dependent re-joining activity.

To understand whether the BIA-induced DSBs were dependent on Topo I function, Topo I was transiently depleted by siRNA transfection, and the BIA-induced formation of DSBs was analysed by PFGE. First, the depletion of Topo I protein through the transfection of siRNA against Topo I was confirmed (Fig. 3a, Supplementary Fig. 6a). The BIA-induced formation of DSBs under Topo I-depleted conditions was then analysed by PFGE. Cells treated with CPT showed strong induction of DSBs, but Topo I depletion reduced the accumulation of DSBs (Fig. 3b,c, Supplementary Fig. 6b). BIA-induced DSB formation was also suppressed by Topo I depletion (Fig. 3b,c). This result indicated that BIA-induced DSB formation was dependent on Topo I function. We then assessed whether Topo I-DNA covalent complexes accumulated after treatment with berberine and coptisine. To this end, we performed an in vivo complex of enzyme (ICE) assay^28^. Cells were treated with CPT, coptisine and berberine, and Topo I-DNA covalent complexes were visualized by immunoblotting using an anti-Topo I antibody. Treatment with CPT induced the accumulation of Topo I-DNA covalent complexes, but neither coptisine nor berberine prompted the accumulation of Topo I-DNA complexes (Fig. 3d, Supplementary Fig. 6c). This result indicates that coptisine and berberine do not stabilize the DNA-Topo I covalent complex in vivo.

**Figure 6:**
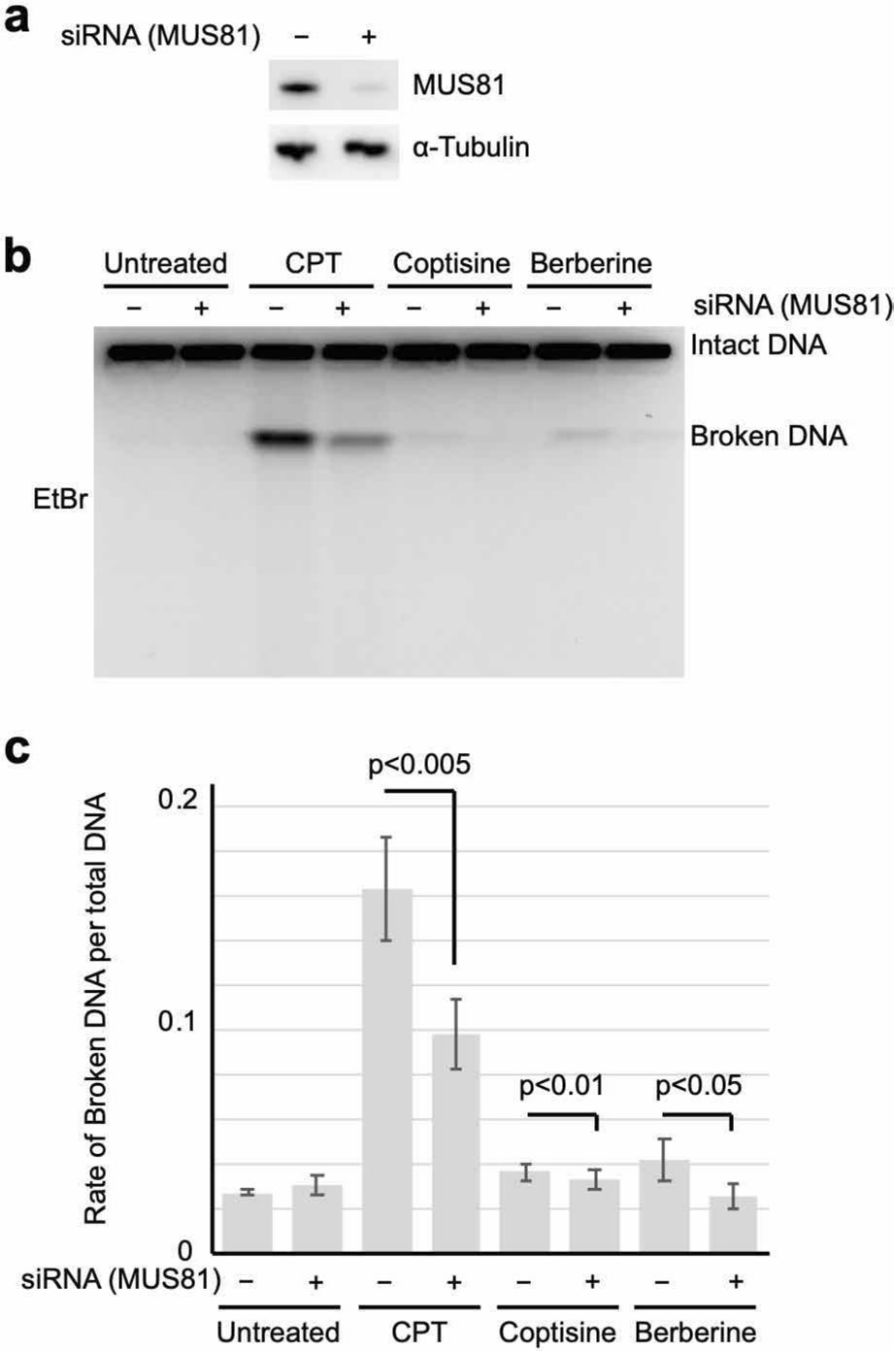
Analysis of MUS81 function on BIA-induced DSB formation in vivo. (**a**) Western blot analysis of MUS81 after transfection with siRNA against the *MSU81* gene. (**b**) PFGE analysis of DSB accumulation after treatment with transient MUS81 depletion by siRNA combined with berberine and coptisine. (**c**) Quantification of broken DNA per total DNA after treatment with transient Topo I depletion by siRNA combined with berberine and coptisine. The means and SEs were determined from four independent experiments.

Based on these results, we concluded that treatment with berberine and coptisine mildly induces Topo I-dependent DSBs, but the action of these compounds on Topo I does not stabilize Topo I-DNA complexes.

### Characterization of topoisomerase inhibition by berberine and coptisine in vitro

Because the BIA-induced DSBs were suppressed by combined treatment with aphidicolin, it is possible that the formation of DSBs induced by these alkaloids depends on topoisomerase inhibition. Indeed, some studies have revealed that berberine analogues could inhibit the activity of Topo I^29^. First, we analysed the inhibitory effects of berberine, coptisine, palmatine, and magnoflorine on Topo I activity. The relaxation activity of Topo I was inhibited by coptisine and berberine and weakly inhibited by palmatine (Fig. 4a,b, Supplementary Fig. 7a,b), whereas magnoflorine exerted no inhibitory effect (Fig. 4c, Supplementary Fig. 7c). These results implicate that the five-membered rings located on both ends of the indicated BIAs could be associated with the inhibitory effect on Topo I.

**Figure 7:**
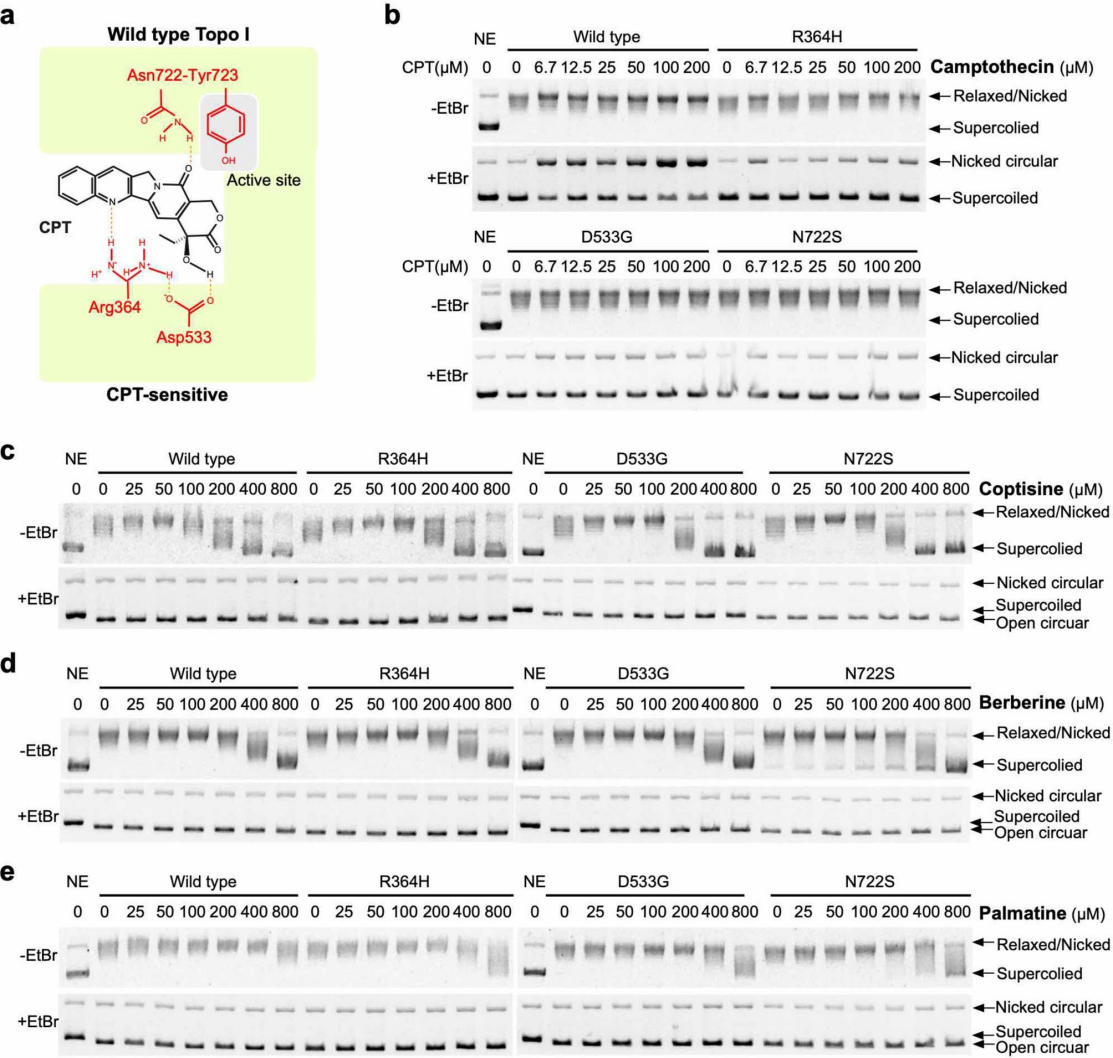
Analysis of the inhibitory effects of BIAs against CPT-resistant Topo I proteins. (**a**) Schematic representation of CPT-resistant *TOP1* mutations. (**b**) Relaxation activity of CPT-resistant Topo I proteins in the presence of CPT in vitro. (**c**) Inhibitory effect of coptisine, berberine, epiberberine and palmatine on CPT-resistant Topo I proteins in vitro.

As mentioned above, the effect of CPT is blocking the DNA rejoining step, which results in accumulation of the DNA-Topo I complex^13^. However, the mechanism through which these BIAs act against Topo I is not yet understood. Therefore, we explored whether the aforementioned alkaloids stabilize the DNA-Topo I complex. The DNA-Topo I complex stabilized during the Topo I reaction was detected as nicked DNA by agarose gel electrophoresis in the presence of 0.5 μg/mL ethidium bromide (EtBr) (Fig. 4d, Supplementary Fig. 7d). Therefore, these BIAs were treated with negatively supercoiled DNA, and Topo I and the DNA-Topo I complex were analysed by EtBr-containing agarose gel electrophoresis (Fig. 4d, Supplementary Fig. 7d). None of these BIAs induced accumulation of the DNA-Topo I complex (Fig. 4d, Supplementary Fig. 7d). This result suggests that berberine and coptisine act as inhibitors of Topo I rather than as stabilizers of the DNA-Topo I complex, which is consistent with the cellular results.

In addition to direct inhibition, the activities of topoisomerase I are significantly affected by substances that can bind to DNA^30,31^. Therefore, we analysed the DNA-binding activities of berberine, coptisine, and palmatine by 1D-^1^H NMR titration experiments^32^. The titration of aliquots of dsDNA (CGATCG)_2_ into BIA solutions induced ^1^H chemical shift changes in the BIA signals in a concentration-dependent manner (Fig. 4e). The 10th proton in the alkaloid backbone was used to generate the binding curves (Fig. 4e). The obtained DNA-binding affinities of these alkaloids were almost comparable (Fig. 4e). This result suggests that the inhibition of Topo I by these BIAs must be a direct effect rather than an indirect effect through the DNA binding of these BIAs.

Because berberine and coptisine can bind to DNA, one possibility is that these alkaloids indirectly inhibit Topo I by intercalating into DNA. To rule out this possibility, we assessed whether these BIAs can inhibit the nicking^33^ and rejoining activity on short oligo-DNAs^34^. The nicking and rejoining activities on short linear DNAs are less effective due to the inhibitory effect of intercalation. First, the inhibitory effect of nicking activity was explored. Because we cannot use any radioisotopes in our facility at this moment, the Cy-3-labelled substrate was used (Fig. 5a). Treatment with CPT induced nicked DNAs, whereas neither berberine nor coptisine induced accumulation of nicked DNAs (Fig. 5b, Supplementary Fig. 8a). This result is consistent with the above-mentioned results. To confirm the inhibitory effect of berberine and coptisine on Topo I, the rejoining activity was also analysed. In this experiment, we assessed the joining activity of 3′-Cy5-labelled DNA on 5′-Cy3-labelled DNA by Topo I (Fig. 5c) and found that Topo I induced rejoining between these two substrates (Fig. 5d). Treatment with berberine and coptisine resulted in an increase in unreacted molecules (18-mer DNAs were detected by Cy-3 labelling) and a decrease in rejoined products (36-mer DNAs were detected by Cy-5 labelling) (Fig. 5d, Supplementary Fig. 8b). These findings suggest that berberine and coptisine inhibit Topo I function at the nicking step.

**Figure 8:**
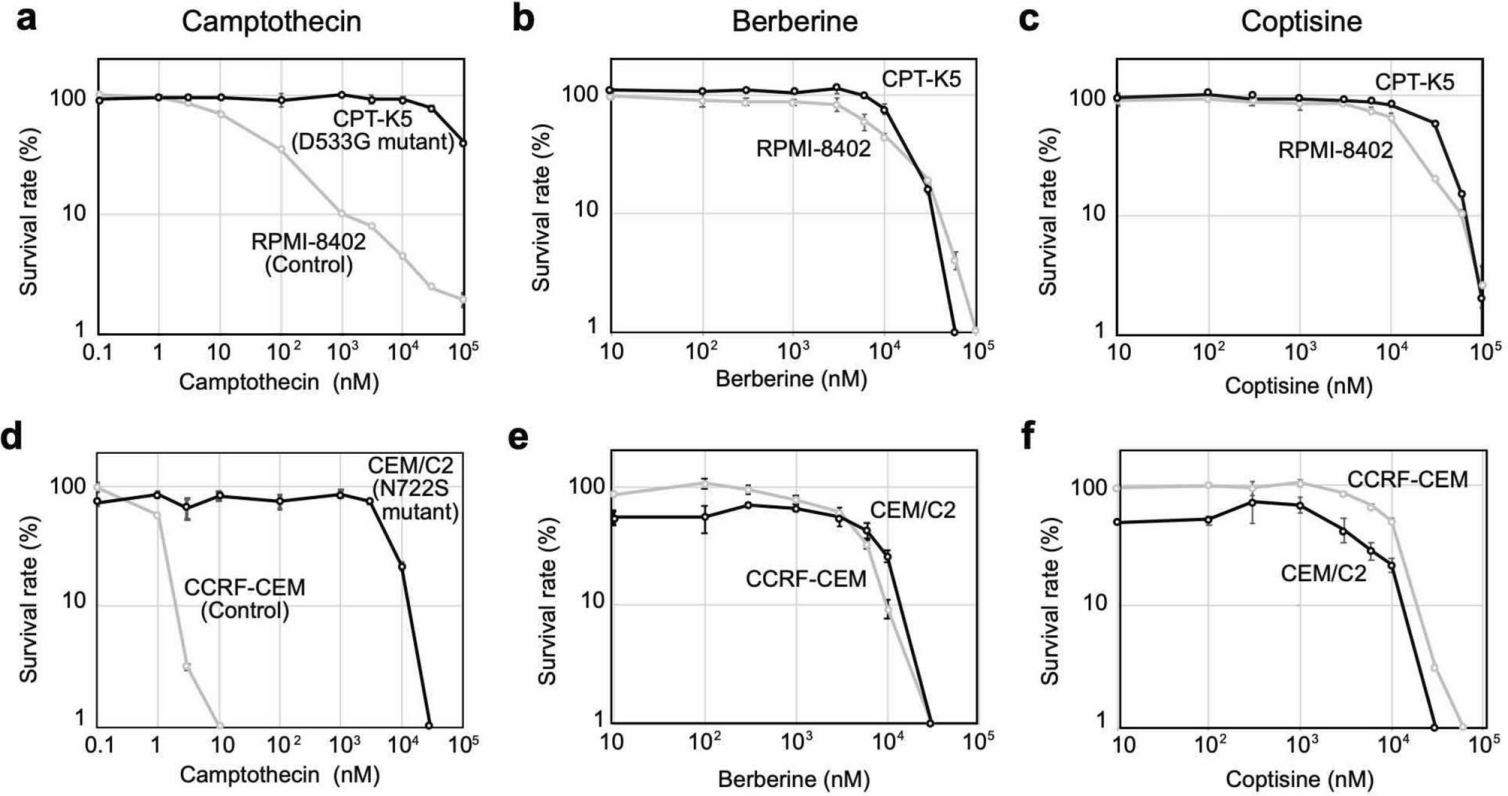
Analysis of the inhibitory effects of BIAs against CPT-resistant cells and Topo I proteins. (**a**) Analysis of CPT resistance of CPT-K5 cells carrying D533G mutations in the *TOP1* gene. (**b,c**) Survival curve of RPMI-8402 and CPT-K5 cells after treatment with berberine (**b**) and coptisine (**c**). (**d**) Analysis of CPT resistance of CEM/C2 cells carrying N722S mutations in the *TOP1* gene. (**e,f**) Survival curve of CCRF-CEM and CEM/C2 cells after treatment with berberine (**e**) and coptisine (**f**).

Based on these results, we determined that berberine and coptisine inhibit the nicking step of Topo I.

### Role of MUS81-EME1 structure-specific endonuclease on berberine- and coptisine-induced DSB formation

Although DSB formation occurs after treatment with berberine and coptisine in vivo (Figs. 1, 2, 3), we could not detect the effect of these BIAs in stabilizing the Topo I-DNA complex in vitro (Figs. 3b,d, 4, 5). Then one question is how DSB occurs after treatment with berberine and coptisine. Previously, Regairaz et al. showed that the DNA replication fork collapsed by CPT was cleaved by MUS81-EME1 structure-specific endonucleases^41^. One possibility is that stalled replication forks must be induced by inactivation of Topo I function after treatment with these BIAs, and such stalled replication forks are cleaved by MUS81-EME1 structure-specific endonucleases. To address this, MUS81 was transiently depleted by siRNA transfection, and the BIA-induced formation of DSBs was analysed by PFGE. First, the depletion of MUS81 protein through the transfection of siRNA against MUS81 was confirmed (Fig. 6a, Supplementary Fig. 9a). The BIA-induced formation of DSBs under MUS81-depleted conditions was then analysed by PFGE. As it shown in the previous study, MUS81 depletion reduced the accumulation of CPT-induced DSBs (Fig. 6b,c, Supplementary Fig. 9b). BIA-induced DSB formation was also suppressed by MUS81 depletion (Fig. 6b,c). This result indicated that BIA-induced DSB formation was dependent on MUS81 function.

**Figure 9:**
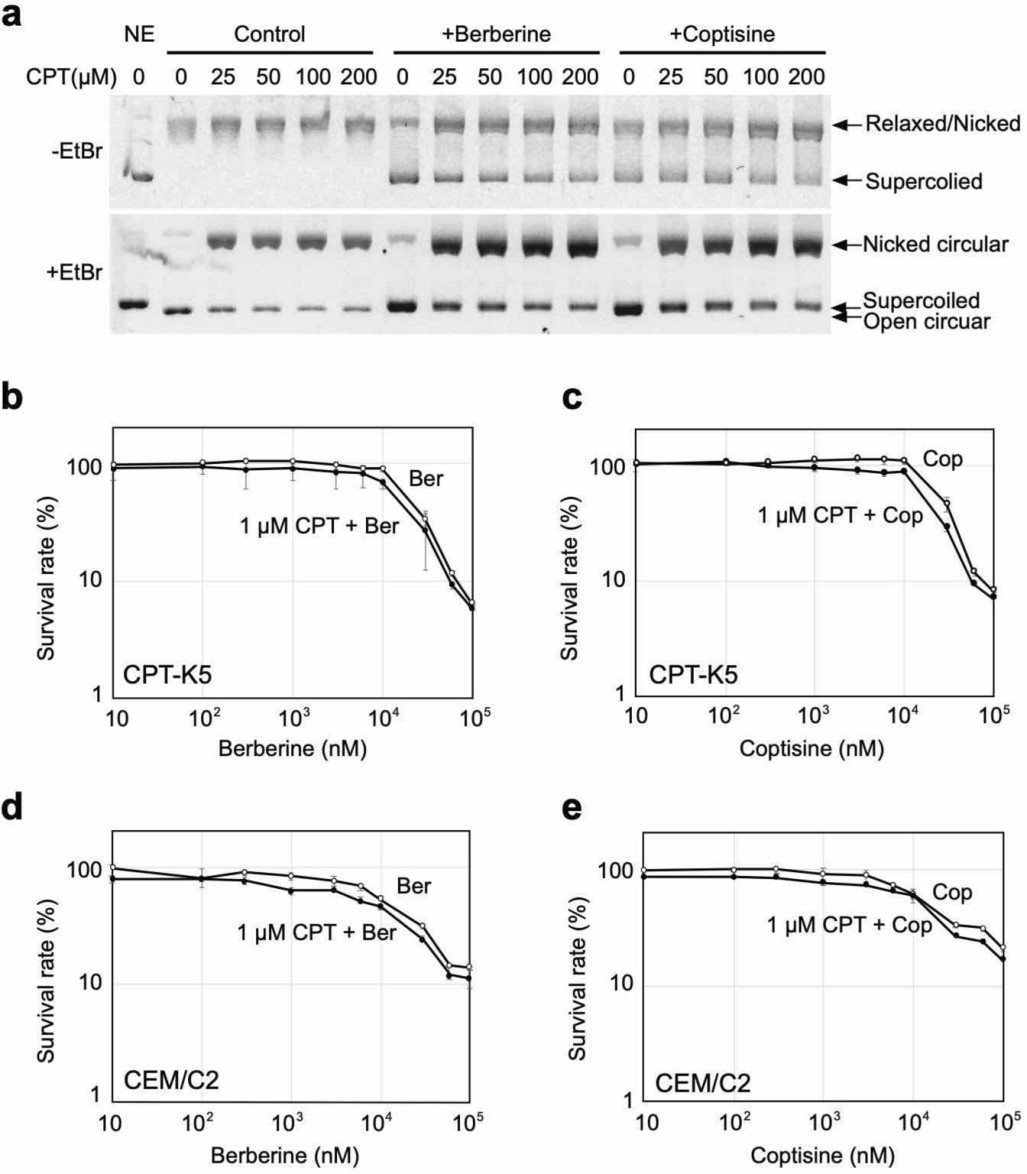
Effect of treatment with CPT combined with berberine and coptisine. (**a**) Effect of treatment with CPT combined with berberine and coptisine on the relaxation assay in vitro. (**b-e**) Effect of treatment with CPT combined with berberine and coptisine on the survival of CPT-resistant cell lines, as determined through the MTT assay. (**b,c**) Effect of treatment with CPT combined with berberine (**b**) and coptisine (**c**) on the survival of the CPT-K5 cell line, as determined through the MTT assay. (**d,e**) Effect of treatment with CPT combined with berberine (**d**) and coptisine (**e**) on the survival of CEM/C2 cells, as determined through the MTT assay.

Based on these, we concluded BIA-induced DSB formation occurs by the action of MUS81-EME1 structure-specific endonucleases on stalled replication forks after treatment with these BIAs.

### Action of berberine and coptisine against CPT-resistant mutants

Comparisons of the structures of CPT and the aforementioned alkaloids revealed that none of the alkaloids possess the branched functional group that CPT utilizes in its interaction with Topo I protein (Fig. 7a, Supplementary Fig. 10). We therefore examined whether these BIAs could inhibit CPT-resistant Topo I proteins in vitro. Recombinant proteins of wild-type Topo I mutations, namely, R364H, D533G, and N722S, were expressed in a baculovirus system and purified (Supplementary Fig. 11a). The activities of the purified wild-type and mutant Topo I proteins were confirmed based on their relaxation activities using commercial untagged Topo I protein (Supplementary Fig. 11b,c). The resistance of the mutant proteins to up to 200 μM CPT was confirmed (Fig. 7b). A concentration of 200 μM was the maximum concentration that we could test because a higher concentration resulted in precipitation in the reaction buffer. In the case of wild-type Topo I, treatment with CPT induced nicked DNA by stabilizing the DNA-Topo I complex, and we were able to confirm this activity. Agarose gel electrophoresis without EtBr (-EtBr gel) showed the accumulation of relaxed/nicked DNA, and agarose gel electrophoresis with EtBr revealed the accumulation of nicked DNA in the presence of CPT at a concentration between 6.7 and 200 μM (+ EtBr gel) (Fig. 7b, Supplementary Fig. 12a). Treatment with CPT could not induce the accumulation of nicked DNA when the R364H, D533G and N722S mutants were used (Fig. 7a, Supplementary Fig. 12a). Thereafter, the effects of the selected BIAs on the relaxation activities of CPT-resistant Topo I proteins were examined. As expected, the aforementioned BIAs inhibited CPT-resistant Topo I proteins in a comparable manner to that obtained with the wild-type Topo I protein (Fig. 7c–e, Supplementary Fig. 12b–d). This result suggests that the inhibition of Topo I by the aforementioned BIAs does not require the Arg364, Asp533, and Asn722 residues of Topo I, which indicates that the binding mode of berberine and coptisine that result in Topo I inhibition is different from that of CPT. The interaction modes of these alkaloids with Topo I differ from that of CPT. We then hypothesized that berberine and coptisine will maintain their cytotoxic activity against cells carrying CPT-resistant *Top1* mutations. To assess this hypothesis, we employed two cell lines that carry the D533G and N722S mutations. As previously mentioned, these mutations result in defects in the interaction between CPT and Topo I. We obtained two CPT-resistant cell lines, namely, CPT-K5 carrying the D533G mutation^16,35^ and CEM/C2 carrying the N722S mutation^16^, as well as their control cell lines, RPMI8204 and CCRF-CEM, respectively (Fig. 8). Because these cell lines are suspension-type cells, the survival curves of CPT and the aforementioned alkaloids were determined by MTT assays. First, we confirmed that CPT-K5 and CEM/C2 cells showed resistance to CPT compared with their control cells (Fig. 8a,d). The CPT-K5 and CEM/C2 cells were then treated with the indicated BIAs, and their survival curves were determined. Both groups of CPT-resistant cells, namely, CPT-K5 and CEM/C2, were killed by BIAs at a dose comparable to that needed to eliminate their control cells (Fig. 8b,c,e,f). These results indicate that the D533G and N722S mutations do not affect the cytotoxic effects of berberine and coptisine, which suggests that berberine and coptisine can remove both CPT-resistant cells and CPT-sensitive cells in a similar manner.

**Figure 10:**
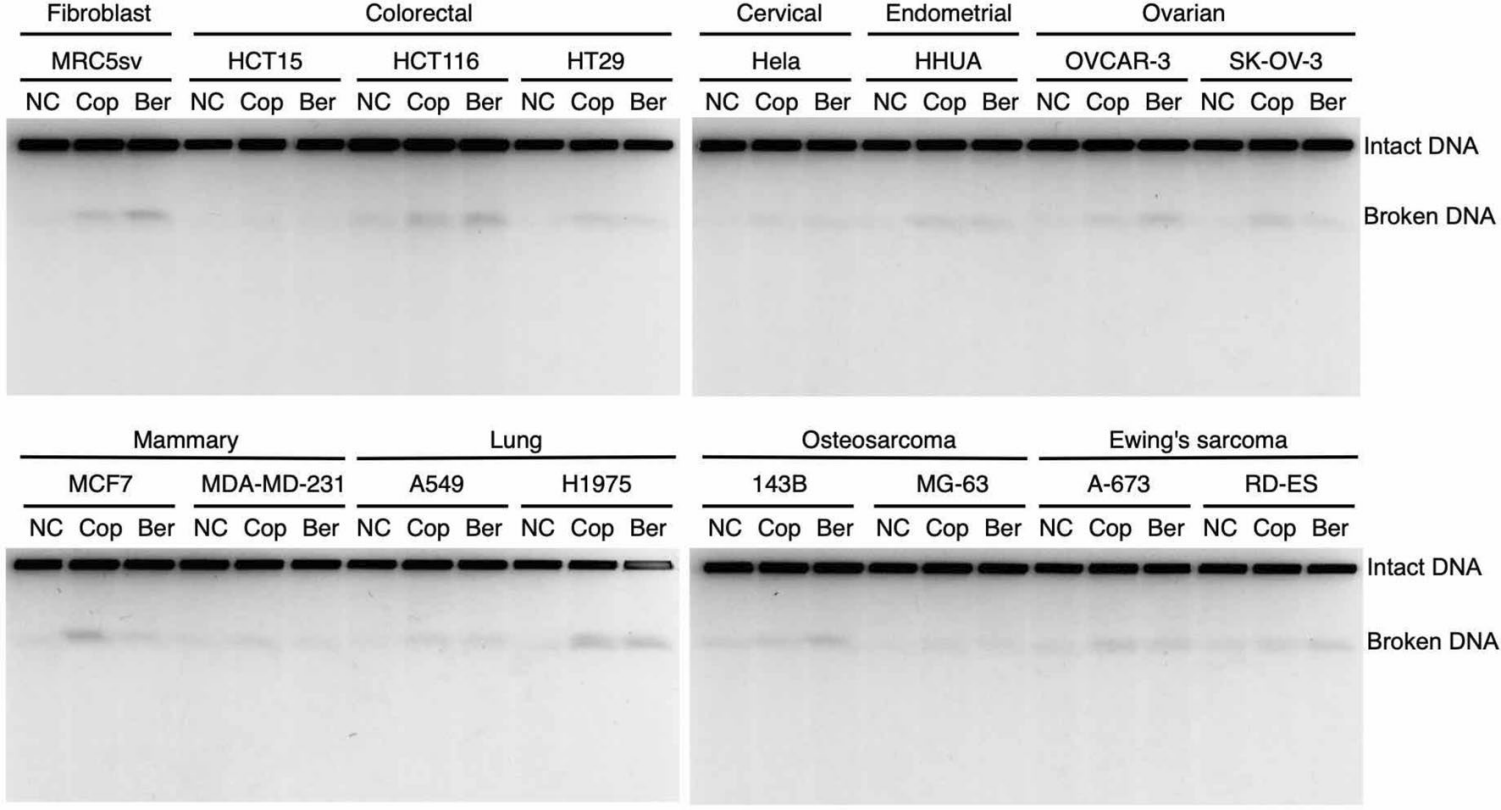
PFGE analysis of DSB accumulation after treatment with berberine and coptisine in various cancer cell lines. Various cancer cell lines were treated with 10 μM coptisine and berberine for 24 h, and the accumulation of DSBs was analysed by PFGE.

Because the effects of berberine and coptisine appear to inhibit the nicking step of Topo I, which is a different step than that inhibited by CPT, we assessed the effects of CPT in combination with these BIAs. First, various concentrations of CPT were added in the presence of berberine or coptisine, and the inhibitory effect of Topo I was analysed by a relaxation assay. After treatment with only CPT (Control), all substrate DNAs migrated at the relaxed and nicked DNA positions in the -EtBr gel, but a small amount of unreacted supercoiled appeared in the + EtBr gel (Fig. 8a). This result indicated that a large amount of DNA was nicked by the action of CPT, but unaffected DNA was relaxed by Topo I. However, after the combined treatments, the majority of the DNA migrated in the nicked DNA position, but some fraction of the DNA was detected as supercoiled DNA (Fig. 9a, Supplementary Fig. 13). This result indicated that CPT introduced nicked DNA and that BIAs inhibited the relaxation activity of Topo I, which suggests that these BIAs independently act on CPT. We subsequently explored the effect of the combined treatments at the cellular level. Using CPT-resistant cell lines (CPT-K5 and CEM-C2), berberine and coptisine were titrated in the presence of 1 μM CPT, which is higher than the 90% viability concentration on these cell lines, and compared the results with those obtained in the absence of CPT. In all cases, the survival rates obtained with the combined treatments were almost comparable or indicated slightly increased sensitive to those obtained with the single BIA treatments (Fig. 9b). These results also indicate that BIAs independently act on CPT and that the action of berberine and coptisine against Topo I is independent of CPT.

Based on these results, we concluded that both berberine and coptisine possess cytotoxic activity independent of CPT and can remove cells carrying CPT-resistant mutations in the *TOP1* gene at a level comparable to that obtained with wild-type CPT-sensitive cells.

### Investigation of BIA-induced DSB formation in various cancer cell lines

To assess whether treatments with berberine and coptisine can induce DSBs in cancer cell lines, we explored the accumulation of DSBs various cancer cell lines after treatment with 10 μM coptisine and berberine for 24 h by PFGE. The following cancer cell lines were tested: colorectal cancer cell lines, HCT15, HCT116, and HT29; cervical cancer cells, HeLa; endometrial cancer cell line, HHUA; ovarian cancer cells, OVCAR-3 and SK-OV-3; mammary cancer cells, MCF7 and MDA-MD-231; lung cancer cells, A549 and H1975; osteosarcoma, 143B and MG-63; and Ewing sarcoma, A-673 and RD-ES. Most cell lines showed a similar tendency to that obtained with the SV40-transformed human fibroblast cell line MRC5sv, and no clear tissue specificity was observed (Fig. 9). However, some cell lines, such as HCT15, MDA-MD-231, and MG-63, exhibited less induction of DSBs (Fig. 10, Supplementary Fig. 14). These results suggest that berberine and coptisine can act in cancer cell lines.

## Discussion

Pharmacologists have attempted to finding efficient methods for fighting diseases, including cancer^1,36^, for a long time, and natural medicines have been used in cancer treatments before modern anticancer agents were developed. To discover additional substances with anticancer activity, we screened selected Japanese herbal medicines, and treatments with two extracts prepared from Phellodendron bark (#8) and Coptis rhizome (#10) induced accumulations of DSBs (Supplementary Fig. 1). The analysis of available data for natural compounds revealed that berberine and palmatine are major components of these herbal medicines, and we subsequently identified berberine and coptisine. We cannot rule out the possibility that other compounds, including these herbal extracts, might have similar activity, but at least in this study, berberine and coptisine could induce DSBs by inhibiting Topo I function (Figs. 1, 2, 3). The cytotoxic activities of berberine and coptisine have previously been reported^26,27^, but we investigated new aspects of these compounds. First, we found that at least one five-membered ring of coptisine and berberine plays a crucial role in the inhibitory effect of Topo I (Figs. 3, 4). It is likely that the conformational flexibility of the methoxy groups affects the interaction between BIA and DNA and/or BIA and Topo I. One possibility is that the DNA-binding activities of these alkaloids are involved in Topo I inhibition. In this study, we found that the DNA-binding affinities of berberine, coptisine, and palmatine were similar (Fig. 3c) and not correlated with Topo I inhibition. Previous studies have suggested two distinct DNA interactions of berberine: one is through intercalation^37,38^, and the other involves what is known as minor groove-directed interaction^39^, where the binding occurs on the minor groove of the DNA and in which the convex side is linked to the helix groove. This finding implies that the DNA-binding mode might be involved in Topo I inhibition. However, Pilch et al. showed that not only intercalations but also minor groove-directed interactions were sufficient to inhibit human Topo I activity^29^. Therefore, it is unlikely that the DNA-binding mode is correlated with the inhibitory effect of berberine and coptisine on Topo I. Based on the aforementioned evidence, we hypothesized that berberine and coptisine block the catalytic function of Topo I by forming a stable interaction at the active site. Previously, the interaction of berberine, but not coptisine, with Topo I was investigated, and the results indicate that berberine is able to bind to the active site of Topo I, which is the same site to which CPT binds^40^. Berberine and coptisine might stably bind to the active site of Topo I, whereas palmatine could be slightly more unstable due to the movements of methoxy groups. Second, we found that the cytotoxic effects of berberine and coptisine against CPT-resistant cancer cells were comparable to those found with the control (CPT-sensitive) cells (Fig. 8). The structural comparisons predicted that the binding modes of berberine and coptisine with Topo I were different from that of CPT (Supplementary Fig. 10), and we confirmed this phenomenon using CPT-resistant proteins and cells (Figs. 7, 8, 9). Based on the results, we identified berberine and coptisine as good candidates for use in combination with CPT to increase the effectiveness of cancer treatment and suppress CPT-resistant cancer cells.

Next, we tried to address the mechanism through which DSB formation occurs after treatment with berberine and coptisine, but we could not fully understand its mechanism. This certainly requires further investigation. First, we examined whether BIA-induced DSB formation was suppressed by cotreatment with aphidicolin. Previous studies have suggested that aphidicolin acts as an antagonist of CPT-induced DSB formation^14^ but not etoposide-induced DSB formation because etoposide has not only S phase-dependent toxicity but also S phase-independent toxicity^24^. BIA-induced DSB formation was largely suppressed by cotreatment with aphidicolin, and BIA-induced DSB formation associated with DNA replication occurred. Next, we accessed if SSB formed by the Topo I-DNA complex might be converted to a DSB through progression of the DNA replication fork or not. However, we could not detect the effect of berberine and coptisine in stabilizing the Topo I-DNA complex (Figs. 3b,d, 4, 5). Therefore, it is unlikely that an SSB formed by the Topo I-DNA complex might be converted to a DSB through progression of the DNA replication fork. Third, we explored the possibility that treatment with berberine and coptisine causes stalled DNA replication forks by inhibiting Topo I, and stalled DNA replication forks were cleaved by MUS81-EME1 structure-specific endonuclease, resulting in the formation of DSBs at DNA replication sites. Indeed, it is shown that the dysfunction of Topo I due to treatment causes stalled replication forks^15^, and the DNA replication fork collapsed by CPT was cleaved by MUS81-EME1 structure-specific endonucleases^41^. Here we showed that BIA-induced DSB formation was dependent on the MUS81 function (Fig. 6). Therefore, it is likely that some stalled replication forks might be cleaved by a MUS81-EME1 structure-specific endonuclease, which results in the formation of DSBs. Forth, we also detected that cells depleting Topo I by siRNA did not show DSB formation (Fig. 3). Since Topo I is essential for DNA replication, the cell without Topo I cannot initiate DNA replication, whose situation is the similar to that treated with aphidicolin. It is likely that Topo I-depletion reduced the number of active DNA replication forks, and this automatically results in the reduction of stalled DNA replication forks by treatment with these BIAs. Thus, cells depleting Topo I by siRNA did not show DSB formation. Based on these, we suggest that stalled replication forks induced by inactivation of Topo I function after treatment with berberine and coptisine must be cleaved by MUS81-EME1 structure-specific endonucleases.

One concern regarding the use of berberine and coptisine in clinical settings is that their cytotoxicity is not sufficiently strong for the compounds to be considered efficient anticancer treatments. An anticancer drug used in chemotherapy has an LD_50_ lower than 1 μM. Therefore, modifying the chemical structure of berberine and coptisine to increase their cytotoxicity is certainly needed. Moreover, we cannot deny the possibility that berberine and coptisine have another target molecule that causes cytotoxicity. Understanding the mechanisms of these alkaloid actions will certainly increase the knowledgebase of the chemical biology field as well as possibly inform improvements in chemotherapy regimens.

Natural medicines prepared using Phellodendron bark and Coptis rhizome are generally administered as gastrointestinal and anti-inflammatory agents. Currently, berberine chloride is an authorized antibacterial drug in Japan used in the treatment of diarrhoea. In contrast, a major side effect of CPT is severe diarrhoea. In Japan and China, natural medicines, such as Hange-shashin-to, Sairei-to, Shengjiang Xiexin decoction, Banxia Xiexin decoction, Huangqin decoction, and PHY906, have been found to be effective in preventing CPT-induced diarrhoea^42^. Interestingly, Coptis rhizome is a common ingredient in all of these medicines^42^. Although the involvement of berberine and coptisine in the suppression of CPT-induced diarrhoea has never been examined, identifying substances that can suppress CPT-induced diarrhoea is certainly important. Overall, a screening of Japanese herbal medicines successfully identified berberine and coptisine as DSB inducers at DNA replication sites, and we found that berberine and coptisine inhibit Topo I, even in CPT-resistant cells, and thereby induce cell death. Natural product screening might be regarded as an obsolete technology; however, there remain many undiscovered substances that might exhibit great potential in healthcare as well as academic research in medicine, chemistry, and biology, particularly with regard to improving chemotherapy regimens and understanding the underlying pharmacological actions of these natural medicines. Acquiring knowledge from nature might be considered old-fashioned but could also lead to a novel discovery.

### Experimental procedures

#### Cell lines and media

All human cells were cultivated at 37 °C in the presence of 5% CO_2_. The SV40-transformed human fibroblast line MRC5sv was cultivated in Dulbecco's modified Eagle’s medium (DMEM) with 10% foetal bovine serum (FBS). The human colorectal carcinoma cell lines HCT15 and HCT116 and the human lung adenocarcinoma cell line H1975 were cultivated in Roswell Park Memorial Institute (RPMI) 1640 medium with 10% FBS. The human colorectal adenocarcinoma cell line HT29, the lung carcinoma cell line A549, the human cervical adenocarcinoma cell line HeLa, the human endometrial adenocarcinoma cell line HHUA, the human ovarian adenocarcinoma cell line SK-OV-3, the human bone osteosarcoma cell lines 143B and MG-63, the human Ewing’s sarcoma cells A-673 and RD-ES, and the human mammary adenocarcinoma cell line MDA-MB-231 were cultured in DMEM with 10% FBS. The human ovarian adenocarcinoma cell line OVCAR-3 was cultivated in RPMI 1640 medium with 0.01 mg/mL bovine insulin and 20% FBS. The human mammary adenocarcinoma cell line MCF-7 was cultivated in DMEM with 0.01 mg/mL human insulin and 10% FBS. The human lymphoblast cell lines RPMI8402, CPT-K5, CCRF-CEM (ATCC CCL-119), and CEM/C2 (ATCC CRL-2264) were cultivated in RPMI 1640 medium with 10% FBS. The OVCAR-3 and CPT-K5 cell lines were purchased from the Japanese Collection of Research Bioresources (JCRB) Cell Bank (Osaka, Japan). The CCRF-CEM (ATCC CCL-119) and CEM/C2 (ATCC CRL-2264) cell lines were obtained from the American Type Culture Collection (ATCC) (Manassas, VA, USA).

#### Immunofluorescent staining

Subconfluent MRC5sv cells on coverslips were treated with 100 μg/mL herbal extract and 100 nM camptothecin for 24 h. Thereafter, the cells were fixed with 2% paraformaldehyde in phosphate-buffered saline (PBS) for 15 min and permeabilized with 0.25% Triton X-100 in PBS. Rabbit polyclonal anti-53BP1 antibody (1:300; Novus Biologicals) and mouse monoclonal anti-γ-H2AX antibody (1:500; Millipore) were used as the primary antibodies. Alexa 488-conjugated goat anti-mouse antibody (1:200; Molecular Probes, Thermo Fisher Scientific) and AF594-conjugated goat anti-mouse antibody (1:200; Molecular Probes, Thermo Fisher Scientific) were used as secondary antibodies. The cells were visualized using a fluorescence microscope (Leica TCS SP8).

#### Pulsed-field gel electrophoresis (PFGE)

Subconfluent cultures of MRC5sv were prelabelled with 10 μM IdU for 30 min, washed with PBS and placed in refreshed medium. After labelling with IdU, the cells were treated with various herbal extracts and alkaloids for 24 h. Thereafter, the cells were harvested by trypsinization, and plugs (0.5% (w/v) agarose containing 10^5^ cells in PBS) were prepared with a CHEF disposable plug mould (BioRad). The plugs were incubated in lysis buffer (100 mM EDTA, 1% (w/v) lauryl sarcosine sodium, 0.2% (w/v) sodium deoxycholate and 1 mg/mL proteinase K) at 37 °C for 24 h and then washed with TE buffer (10 mM Tris–HCl (pH 8.0) and 100 mM EDTA). PFGE was performed at 13 °C for 23 h in 0.9% (w/v) agarose containing 0.25X TBE buffer using a Biometra Rotaphor (Analytik Jena). The parameters were as follows: voltage between 180 and 120 V, linear angles from 120 °C to 110 °C, and interval lengths of 30 s to 5 s. The gel was stained with ethidium bromide (EtBr) and analysed using a Typhoon FLA7000 scanner (GE Healthcare). Semiquantitative analysis was performed using ImageQuant (GE Healthcare)^43^.

#### Immunoblotting of IdU-labelled DNAs

After analysis of the EtBr-stained gel, the bottom half of the gel was removed. The upper part of the gel containing intact DNA and broken DNA was exposed to 2-kJ/m^2^ UV light (254 nm). Thereafter, the gels were treated with denaturation buffer (0.5 N NaOH and 1.5 M NaCl) for 1 h and then with neutralization buffer (1 M Tris–Cl (pH 7.6) and 1.5 M NaCl) for 1 h. The DNA was then transferred to a Hybond-N + membrane (GE healthcare) with 20X SSC buffer (3 M NaCl and 0.3 M trisodium citrate) overnight. The transferred DNA on the membrane was crosslinked using a UV crosslinker (1.2 kJ/m^2^) (UVP). The membranes were then treated with blocking buffer (5% skim milk and 0.1% Tween 20 in PBS) for 1 h. After blocking, mouse anti-BrdU antibody (1:5000, BU-48, BD) or mouse anti-BrdU antibody (1:5000, B-33, Sigma) was added to the blocking buffer, and the membranes were incubated for 3 h. The membranes were then subjected to three 10-min washes with 0.1% Tween 20 in PBS. The alkaline phosphatase (AP)-conjugated donkey anti-mouse antibody (1:10,000, Jackson) and 0.1% Tween 20 in PBS were added to the membranes, and the membranes were incubated for 1 h. The membranes were then subjected to three 10-min washes with 0.1% Tween 20 in PBS. Thereafter, the signals were visualized with BCIP/NBT solution (Roche) in AP buffer (0.1 M Tris–Cl (pH 9.5), 100 mM NaCl and 50 mM MgCl).

#### Herbal extracts and compounds

Herbal medicines were originally imported from China to Japan; however, Japanese herbal medicines were prepared using Japanese formulas. The herbal extracts used in this study were provided by the Institute of Natural Medicine, University of Toyama, Japan. Detailed information related to the extracts and compounds is provided in Table S1. Berberine chloride, palmatine chloride, magnoflorine iodide, and camptothecin were purchased from WAKO Co., Ltd. (Osaka, Japan). Coptisine chloride was purchased from Nagara Science Co., Ltd. (Gifu, Japan).

#### Colony survival assay

Five hundred MRC5sv cells were plated in 60-mm dishes. After overnight incubation, the cells were treated with the indicated compounds for 24 h. Subsequently, the cells were washed with PBS and incubated with fresh DMEM with 10% FBS for 10–14 days. The cells were fixed with cell fixation buffer and stained. The means and standard deviations were determined from triplicate data.

#### siRNA and western blotting

Commercial Topo 1 siRNA was used in this study (human Topo1 siRNA, sc-36694, Santa Cruz Biotechnology). Topo I was analysed by western blotting using a mouse anti-Topo I antibody (C-21) (1:10,000, sc-32736, Santa Cruz Biotechnology). Commercial MUS81 siRNA was used in this study (human MUS81 siRNA, L-016143–01-0005, Horizon). MUS81 was analysed by western blotting using a mouse anti-MUS81 antibody (MTA30 2G10/3) (1:10,000, ab-14387, Abcam). As a loading control, α-tubulin was analysed with mouse anti-α-tubulin antibody (1:10,000,10G10 cat# 017–25,031, Fujifilm). HRP-conjugated donkey anti-mouse IgG (1:10,000, 715–035-150, Jackson ImmunoResearch Laboratories) and HRP-conjugated anti-rabbit IgG (1:10,000, 711–035-152, Jackson ImmunoResearch Laboratories) were used as secondary antibodies, and the bands were visualized with Chemi-Lumi One Super (Nakarai Tesque) and analysed with LAS4000 Mini (GE Healthcare Life Science).

#### ICE assay

The ICE assay was performed using an ICE assay kit (TopoGEN). In accordance with the manufacturer’s protocol, the cells were lysed, and the DNA-Topo I complex was purified. The DNA-Topo I complex was transferred to a Hi-bond N + membrane (GE Healthcare) using a slot blot system (BIORAD), and the transferred DNA-Topo I complex on the membrane was crosslinked using a UV crosslinker (1.2 kJ/m^2^) (UVP). The membranes were then treated with blocking buffer (5% skim milk and 0.1% Tween 20 in PBS) for 1 h, stained with mouse anti-Topo I antibody (1:1,000, TG1020-1, TopoGEN) for 1 h, washed three times with 0.1% Tween 20 in PBS and treated with horseradish peroxidase (HRP)-conjugated donkey anti-mouse antibody (Jackson ImmunoResearch Laboratories) for 1 h. The membranes were washed three times with 0.1% Tween 20 in PBS, and the signals were ten visualized with Chemi-Lumi One L (Nakarai Tesque) and analysed using LAS4000 Mini (GE Healthcare).

#### MTT assay

The proliferation activity of the human lymphoblast cell lines RPMI8402, CPT-K5, CCRF-CEM, and CEM/C2 was determined in triplicate using Cell Proliferation Kit I (Roche) following the manufacturer’s instructions. Briefly, two pairs of the cell lines, CEM-C2/CCRF-CEM, and RPMI8402/CPT-K5, were seeded into 96-well plates at a density of 1 × 10^5^ cells/well and incubated at 37 °C with 5% CO_2_ for 1 h. Subsequently, 50 μL of CPT, berberine, coptisine, or epiberberine was added to each well. After 72 h of incubation, 10 μL of the MTT (3-[4,5-dimethylthiazol-2-yl]-2,5-diphenyltetrazolium bromide) labelling reagent was added to each well, and the plate was incubated for 4 h. Thereafter, 100 μL of the solubilization solution was added, and the plates were maintained overnight in the incubator. A cell proliferation curve was then created from the results of the baseline correction absorbance measurements at 570 and 650 nm.

#### Purification of Topo I

His-tagged Topo I was purified using a baculovirus insect cell expression system. The activity of the 6XHis-tagged protein was previously shown^44^. The baculovirus, which carried wild-type, R364H-, D553G-, or N722S-mutated Topo I, was expressed by infecting High Five cells and then incubating the cells for 48 h. After induction, the cells were collected in a 50-mL tube and lysed using lysis buffer (50 mM sodium phosphate buffer (pH 7.0), 1 M NaCl, 1 mM β-mercaptoethanol, and 0.1% Nonidet P-40 with a complete tablet containing a cocktail of inhibitor proteinases) on ice for 1 h. The lysate was clarified by centrifugation (Beckman; 70.1 Ti) at 60,000 X *g* and 4 °C for 1 h, and the supernatant was subsequently incubated with TALON resin (GE healthcare) for 3 h. After incubation, the resin was transferred to a disposable column (BioRad) and washed with 10 bed volumes of HS wash buffer (50 mM sodium phosphate buffer (pH 7.0), 800 mM NaCl, 10% glycerol and 1 mM β-mercaptoethanol) and IM wash buffer (50 mM sodium phosphate buffer (pH 7.4), 500 mM NaCl, 10% glycerol, 1 mM β-mercaptoethanol and 10 mM imidazole). The bound proteins were eluted with elution buffer (50 mM sodium phosphate buffer (pH 7.0), 20 mM glutathione, 300 mM NaCl, 10% glycerol and 1 mM β-mercaptoethanol), and the elution fraction was then loaded on a HiTrap Heparin HP column (GE healthcare) with buffer A (25 mM HEPES-Na (pH 7.5), 150 mM NaCl, 5 mM β-mercaptoethanol and 10% glycerol). The bound protein was eluted in buffer B (25 mM HEPES-Na (pH 7.5), 1 M NaCl, 5 mM β-mercaptoethanol and 10% glycerol) with a linear gradient of 0.15 to 1 M NaCl. The purified protein was confirmed by SDS-PAGE (Supplementary Fig. 11).

#### Relaxation assay

A relaxation assay was performed using Topo I purchased from Takara Co. Ltd. (Fig. 4) and with purified Topo I (Fig. 11b). The purified Topo I was diluted based on its activities with dilution buffer (50 mM HEPES-Na (pH 7.5), 25% glycerol and 5 mM β-mercaptoethanol) (Supplementary Fig. 11). Subsequently, 0.25 μg of supercoiled pBR322 DNA in 10 μL was relaxed with Topo I in reaction buffer (35 mM Tris–Cl (pH 8.0), 72 mM KCl, 5 mM MgCl_2_, 5 mM DTT, 5 mM spermidine and 0.01% BSA). The indicated compound was also added, and the mixture was incubated at 37 °C for 15 min. To stop the reaction, 1/10 vol of 1 mg/mL proteinase K was added, and the mixture was incubated at 37 °C for 5 min. After the reaction, the DNA was analysed by 0.7% agarose gel electrophoresis with TBE buffer in the absence of EtBr. Under this condition, the upper band represents relaxed circular DNA and/or nicked circular DNA, and the intermediate and lower bands represent the supercoiled DNAs. After electrophoresis, the gel was stained with EtBr and analysed using a Typhoon FLA7000 scanner (GE Healthcare). Nicked DNA was analysed by 0.7% agarose gel electrophoresis with TBE buffer in the presence of 0.5 μg/mL EtBr. Under this condition, the upper band represents nicked circular DNA, and the lower band represents covalently closed circular DNA (cccDNA).

#### Nicking and rejoining assay

All oligonucleotides were synthesized (TSUKUBA OLIGO SERVICE). TOP1-Oligo-1-Cy-3 (5′-AAA AAG ACT TGG AAA AAT TTT T -Cy-3–3′) and TOP1-Oligo-1c (5′-AAA AAT TTT TCC AAG TCT TTT T-3′) were used for the nicking assay^33^. The substrate DNA was prepared by annealing TOP-Oligo-1-Cy-3 and TOP1-Oligo-2. Subsequently, 0.25 μM substrate in 10 μL was reacted with Topo I and the indicated compounds (CPT: 100 μM, berberine: 100 μM and 1000 μM, coptisine: 100 μM and 1000 μM). After the addition of loading buffer (0.025% Orange G in 50% glycerol), the samples were incubated at 37 °C for 5 min and at 98 °C for 3 min. After the reaction, the oligo DNA was analysed by 16% urea-polyacrylamide gel electrophoresis with 1X TBE buffer. Under this condition, the upper band was the substrate (36-mer), and the lower band in CPT represented nicked products (22-mer).

For the rejoining assay, the substrate was prepared by annealing TOP1-Oligo-2 and TOP1-oligo-3-Cy-3 (5′-Cy-3-GAT CTA AAA CCC TTG GAA-3′). Subsequently, 0.125 μM substrate in 10 μL was reacted with 1 μM TOP1-Oligo-4 (5′-GGA AAA ATT TTT AAA AAA GAT C-Cy-5–3′), Topo I and the indicated compounds (CPT: 10 μM and 100 μM, berberine: 100 μM and 1000 μM, coptisine: 100 μM and 1000 μM). After the addition of loading buffer, the samples were incubated at 37 °C for 5 min and at 98 °C for 3 min. After the reaction, the oligo DNA was analysed by 16% urea-polyacrylamide gel electrophoresis with TBE buffer. Under this condition, the upper bands that appeared in both the Cy-3 and Cy-5 detections were rejoined products (36-mer), and the lowest bands that appeared only in the Cy-3 detection (18-mer) were unreacted substrates.

After electrophoresis, the gel was analysed using a Typhoon FLA7000 scanner (GE Healthcare).

#### NMR analysis

NMR spectra were recorded with a Bruker Avance600 spectrometer equipped with a TXI cryoprobe at 283 K. 1D ^1^H NMR spectra were acquired using the 3-9-19 watergate pulse sequence^45^. Berberine chloride, coptisine chloride, and palmatine chloride were dissolved in 10 mM sodium phosphate buffer (pH 6.7) in 100% D_2_O. The pD is the pH metre reading uncorrected for the deuterium isotope effect. The concentrations of BIAs were determined spectrophotometrically using the molar absorption coefficients^46,47^: berberine chloride, 22,500 M^−1^ cm^−1^ at 344 nm; coptisine chloride, 19,000 M^−1^ cm^−1^ at 356 nm; and palmatine chloride, 25,000 M^−1^ cm^−1^ at 344 nm. The ^1^H resonance assignments of the BIAs were transferred from a previous study^32^. Aliquots containing 6-mer dsDNA (CGATCG) were added to the BIA solutions (40 μM) in a stepwise manner at BIA:dsDNA molar ratios of 1:0, 1:0.25, 1:0.5, 1:0.75, 1:1, 1:1.5, 1:2, 1:3, 1:5, 1:10, and 1:20. The titration curves were analysed with the program xcrvfit (ver. 4.0.12, http://www.bionmr.ualberta.ca/bds/software/xcrvfit/) to calculate the dissociation constants.

## Supplementary Information

Supplementary Information.

## References

[1] Yu F (2006). Traditional Chinese medicine and Kampo: a review from the distant past for the future. J. Int. Med. Res..

[2] Arita M (2011). Database for crude drugs and Kampo medicine. Genome Inform..

[3] Ouyang H (2011). Multimodality treatment of pancreatic cancer with liver metastases using chemotherapy, radiation therapy, and/or Chinese herbal medicine. Pancreas.

[4] Efferth T, Miyachi H, Bartsch H (2007). Pharmacogenomics of a traditional Japanese herbal medicine (Kampo) for cancer therapy. Cancer Genomics Proteomics.

[5] Pommier Y, Leo E, Zhang H, Marchand C (2010). DNA topoisomerases and their poisoning by anticancer and antibacterial drugs. Chem. Biol..

[6] Kawashima Y (2017). Detection of DNA double-strand breaks by pulsed-field gel electrophoresis. Genes Cells.

[7] Hashimoto S, Anai H, Hanada K (2016). Mechanisms of interstrand DNA crosslink repair and human disorders. Genes Environ..

[8] Hanada K (2006). The structure-specific endonuclease Mus81-Eme1 promotes conversion of interstrand DNA crosslinks into double-strands breaks. EMBO J..

[9] Rowinsky EK, Donehower RC (1995). Paclitaxel (taxol). N. Engl. J. Med..

[10] Steinmetz MO, Prota AE (2018). Microtubule-targeting agents: strategies to hijack the cytoskeleton. Trends Cell Biol..

[11] Sirikantaramas S, Yamazaki M, Saito K (2008). Mutations in topoisomerase I as a self-resistance mechanism coevolved with the production of the anticancer alkaloid camptothecin in plants. Proc. Natl. Acad. Sci. U. S. A..

[12] Pommier Y, Cushman M (2009). The indenoisoquinoline noncamptothecin topoisomerase I inhibitors: update and perspectives. Mol. Cancer Ther..

[13] Staker BL (2002). The mechanism of topoisomerase I poisoning by a camptothecin analog. Proc. Natl. Acad. Sci. U. S. A..

[14] Ryan AJ, Squires S, Strutt HL, Johnson RT (1991). Camptothecin cytotoxicity in mammalian cells is associated with the induction of persistent double strand breaks in replicating DNA. Nucleic Acids Res..

[15] Koster DA, Palle K, Bot ES, Bjornsti MA, Dekker NH (2007). Antitumour drugs impede DNA uncoiling by topoisomerase I. Nature.

[16] Urasaki Y (2001). Use of camptothecin-resistant mammalian cell lines to evaluate the role of topoisomerase I in the antiproliferative activity of the indolocarbazole, NB-506, and its topoisomerase I binding site. Cancer Res..

[17] Chillemi G (2008). Thr729 in human topoisomerase I modulates anti-cancer drug resistance by altering protein domain communications as suggested by molecular dynamics simulations. Nucleic Acids Res..

[18] Mulholland K, Wu C (2016). Computational study of anticancer drug resistance caused by 10 topisomerase I mutations, including 7 camptothecin analogs and lucanthone. J. Chem. Inf. Model..

[19] Arakawa Y, Ozaki K, Okawa Y, Yamada H (2013). Three missense mutations of DNA topoisomerase I in highly camptothecin-resistant colon cancer cell sublines. Oncol. Rep..

[20] Lee YC (2015). Targeting of topoisomerase I for prognoses and therapeutics of camptothecin-resistant ovarian cancer. PLoS ONE.

[21] Teshima R (2020). Investigation of DNA double-strand breaks induced in host cells following infection with genotoxic bacteria. Methods Mol. Biol..

[22] Terabayashi T, Tokumaru A, Ishizaki T, Hanada K (2020). Analysis of chromosomal DNA fragmentation in apoptosis by pulsed-field gel electrophoresis. Methods Mol. Biol..

[23] Cicero AF, Baggioni A (2016). Berberine and its role in chronic disease. Adv. Exp. Med. Biol..

[24] Hawtin RE (2010). Homologous recombination repair is essential for repair of vosaroxin-induced DNA double-strand breaks. Oncotarget.

[25] Shimizu T, Pommier Y (1997). Camptothecin-induced apoptosis in p53-null human leukemia HL60 cells and their isolated nuclei: effects of the protease inhibitors Z-VAD-fmk and dichloroisocoumarin suggest an involvement of both caspases and serine proteases. Leukemia.

[26] Lin CC, Ng LT, Hsu FF, Shieh DE, Chiang LC (2004). Cytotoxic effects of *Coptis chinensis* and *Epimedium sagittatum* extracts and their major constituents (berberine, coptisine and icariin) on hepatoma and leukaemia cell growth. Clin. Exp. Pharmacol. Physiol..

[27] Kobayashi Y (1995). Inhibitors of DNA topoisomerase I and II isolated from the Coptis rhizomes. Planta Med..

[28] Subramanian D, Kraut E, Staubus A, Young DC, Muller MT (1995). Analysis of topoisomerase I/DNA complexes in patients administered topotecan. Cancer Res..

[29] Pilch DS (1997). Minor groove-directed and intercalative ligand-DNA interactions in the poisoning of human DNA topoisomerase I by protoberberine analogs. Biochemistry.

[30] Besterman JM, Elwell LP, Cragoe EJ, Andrews CW, Cory M (1989). DNA intercalation and inhibition of topoisomerase II. Structure-activity relationships for a series of amiloride analogs. J. Biol. Chem..

[31] Montaner B (2005). DNA interaction and dual topoisomerase I and II inhibition properties of the anti-tumor drug prodigiosin. Toxicol. Sci..

[32] Tripathi AN, Chauhan L, Thankachan PP, Barthwal R (2007). Quantum chemical and nuclear magnetic resonance spectral studies on molecular properties and electronic structure of berberine and berberrubine. Magn. Reson. Chem..

[33] Sooryakumar D, Dexheimer TS, Teicher BA, Pommier Y (2011). Molecular and cellular pharmacology of the novel noncamptothecin topoisomerase I inhibitor Genz-644282. Mol. Cancer Ther..

[34] Montaudon D (2007). Inhibition of topoisomerase I cleavage activity by thiol-reactive compounds: importance of vicinal cysteines 504 and 505. J. Biol. Chem..

[35] Kjeldsen E (2018). Characterization of Camptothecin-induced genomic changes in the Camptothecin-resistant T-ALL-derived cell line CPT-K5. Cancer Genom. Proteom..

[36] Yuan H, Ma Q, Ye L, Piao G (2016). The traditional medicine and modern medicine from natural products. Molecules.

[37] Li XL, Hu YJ, Wang H, Yu BQ, Yue HL (2012). Molecular spectroscopy evidence of berberine binding to DNA: comparative binding and thermodynamic profile of intercalation. Biomacromol.

[38] Hill GM, Moriarity DM, Setzer WN (2011). Attenuation of cytotoxic natural product DNA intercalating agents by caffeine. Sci. Pharm..

[39] Mazzini S, Bellucci MC, Mondelli R (2003). Mode of binding of the cytotoxic alkaloid berberine with the double helix oligonucleotide d(AAGAATTCTT)(2). Bioorg. Med. Chem..

[40] Kettmann V, Kost'alova D, Holtje HD (2004). Human topoisomerase I poisoning: docking protoberberines into a structure-based binding site model. J. Comput. Aided Mol. Des..

[41] Regairaz M (2011). Mus81-mediated DNA cleavage resolves replication forks stalled by topoisomerase I-DNA complexes. J. Cell. Biol..

[42] Tang L (2019). Herbal medicines for irinotecan-induced diarrhea. Front. Pharmacol..

[43] Hanada K (2007). The structure-specific endonuclease Mus81 contributes to replication restart by generating double-strand DNA breaks. Nat. Struct. Mol. Biol..

[44] Spanou E (2017). Genetic variability as a regulator of TLR4 and NOD signaling in response to bacterial driven DNA damage response (DDR) and inflammation: focus on the gastrointestinal (GI) tract. Front. Genet..

[45] Liu M (1998). Improved WATERGATE pulse sequences for solvent suppression in NMR spectroscopy. J. Magn. Reson..

[46] Maiti M, Kumar GS (2010). Polymorphic nucleic acid binding of bioactive isoquinoline alkaloids and their role in cancer. J. Nucleic Acids.

[47] Zsila F, Kaman J, Boganyi B, Jozsvai D (2011). Binding of alkaloids into the S1 specificity pocket of alpha-chymotrypsin: evidence from induced circular dichroism spectra. Org. Biomol. Chem..

