# Supplementary material for: The benzylisoquinoline alkaloids, berberine and coptisine, act against camptothecin-resistant topoisomerase I mutants
Source: Sci Rep. 2021 Apr 8;11:7718. doi: 10.1038/s41598-021-87344-2 (PMC8032691; doi:10.1038/s41598-021-87344-2)
Supplement: Supplementary file 1 — Supplementary Information. [file 41598_2021_87344_MOESM1_ESM.pdf]

Supplementary Information

The benzylisoquinoline alkaloids, berberine and coptisine, act against camptothecin-resistant topoisomerase I mutants

Naomi Inoue[1#], Takeshi Terabayashi[2#], Yuri Takiguchi-Kawashima[3†], Daisuke Fujinami[4], Shigeru Matsuoka[5], Masanori Kawano[6], Kazuhiro Tanaka[6], Hiroshi Tsumura[6], Toshimasa Ishizaki[2], Hisashi Narahara[1], Daisuke Kohda[4], Yoshihiro Nishida[1], Katsuhiro Hanada[3*].

[1] Department of Obstetrics and Gynecology, Faculty of Medicine, Oita University, Yufu, Japan.
[2] Department of Pharmacology, Faculty of Medicine, Oita University, Yufu, Japan.
[3] Clinical Engineering Research Center, Faculty of Medicine, Oita University, Yufu, Japan.
[4] Division of Structural Biology, Medical Institute of Bioregulation, Kyushu University, Fukuoka, Japan
[5] Department of Clinical Biology ant Therapeutics, Faculty of Medicine, Oita University, Yufu, Japan.
[6] Department of Orthopaedic Surgery, Faculty of Medicine, Oita University, Yufu, Japan.

*Corresponding authors: Katsuhiro Hanada
Clinical Engineering Research Center, Faculty of Medicine, Oita University, 1-1 Idaigaoka, Hasama-machi, Yufu, Oita 879-5593, Japan
Phone: +81 97 586 5144, Fax: +81 97 586 5146, e-mail: hanada@oita-u.ac.jp

Correspondence can be also sent to Yoshihiro Nishida.
Department of Obstetrics and Gynecology, Faculty of Medicine, Oita University, 1-1 Idaigaoka, Hasama-machi, Yufu, Oita 879-5593, Japan
Phone: +81 97 586 5922, Fax: +81 97 586 6687, e-mail: ynishida@oita-u.ac.jp

[#] These authors contributed equally to this work.

Supplementary Table 1. Herbal extracts used in this study

ID #	Name of herbal medicine	Botanical source
#1	Clematis Root	*Clematis chinensis Osbeck,*<br>*Clematis mandshurica Ruprecht ,*<br>*Clematis hexapetala Pallas*
#2	Artemisia Capillaris Flower	*Artemisia capillaris Thunberg*
#3	Turmeric	*Curcuma longa L.*
#4	Lindera Root	*Lindera strychnifolia (Siebold et Zucc.) F. Vill.*
#5	Corydalis Tuber	*Corydalis turtschaninovii Besser forma yanhusuo Y.H.Chou et C.C.Hsu*
#6	Astragalus Root	*Astragalus membranaceus Bunge,*<br>*Astragalus mongholicus Bunge*
#7	Scutellaria Root	*Scutellaria baicalensis Georgi*
#8	Phellodendron Bark	*Phellodendron amurense Ruprecht*
#9	Cherry Bark	*Prunus jamasakura Siebold ex Koidzumi,*<br>*Prunus verecunda Koehne*
#10	Coptis Rhizome	*Coptis japonica Makino var. japonica Satake,*<br>*Coptis japonica Makino var. dissecta Nakai,*<br>*Coptis japonica Makino var. major Satake,*<br>*Coptis teeta ,*<br>*Coptis chinensis ,*<br>*Coptis deltoidea*
#11	Polygala Root	*Polygala tenuifolia Willdenow*
#12	Artemisia Leaf	*Artemisia princeps Pampanini,*<br>*Artemisia montana Pampanini*
#13	Polygonum Root	*Polygonum multiflorum Thunberg*
#14	Zedoary	*Curcuma zedoaria (Christ.) Roscoe*
#15	Pueraria Root	*Pueraria lobata (Willd.) Ohwi*
#16	Trichosanthes Seed	*Trichosanthes bracteata (Lam.) Voigt*
#17	Processed Ginger	*Zingiber officinale Roscoe*
#18	Glycyrrhiza	*Glycyrrhiza uralensis Fisher,*<br>*Glycyrrhiza glabra Linne*
#19	Platycodon Root	*Platycodon glandiflorum (Jacq.) A. DC.*
#20	Chrysanthemum Flower	*Chrysanthemum morifolium Ramatulle,*<br>*Chrysanthemum indicum Linné*
#21	Immature Orange	*Citrus aurantium Linné var. daidai Makino,*<br>*Citrus aurantium Linné ,*<br>*Citrus natsudaidai Hayata*

#22	Notopterygium	*Notopterygium incisum Ting ex H. T. Chang ,*<br>*Notopterygium forbesii Boissieu*
#23	Apricot Kernel	*Prunus armeniaca Linné,*<br>*Prunus armeniaca Linné var. ansu Maximowicz,*<br>*Prunus sibirica Linné*
#24	Sophora Root	*Sophora flavescens Aiton*
#25	Sasa Leaf	*Sasa palmata Nakai,*<br>*Sasa senanensis Rehder,*<br>*Sasa yahikoensis Makino*
#26	Schizonepeta Spike	*Schizonepeta tenuifolia Briq.*
#27	Suberect Spatholobus Stem	*Spatholobus suberectus Dunn,*<br>*Mucuna birdwoodiana Tutcher*
#28	Cinnamon Bark	*Cinnamomum cassia Blume*
#29	Figwort Root	*Scrophularia ningpoensis Hemsley,*<br>*Scrophularia buergeriana Miquel*
#30	Safflower	*Carthamus tinctorius L.*
#31	Silktree Albizia Bark	*Albizia julibrissin Durazzini*
#32	Red Ginseng	*Panax ginseng C. A. Mey.*
#33	Cyperus Rhizome	*Cyperus rotundus Linné*
#34	Magnolia Bark	*Magnolia obovata Thunb.*
#35	Achyranthes Root	*Achyranthes fauriei Lév. et Vaniot,*<br>*Achyranthes bidentata Blume*
#36	Evodia Fruit	*Evodia rutaecarpa (Juss.) Benth.*
#37	Burdock Fruit	*Arctium lappa Linné*
#38	Sesame	*Sesamum indicum Linné*
#39	Scisandra Fruit	*Schisandra chinensis (Turcz.) Baill.*
#40	Bupleurum Root	*Bupleurum falcatum L.*
#41	Asiasarum Root	*Asiasarum sieboldii (Miq.) F. Maekawa = Asarum sieboldii Miq. ,*<br>*Asiasarum heterotropoides (F. Schmidt) F. Maekawa var. mandshuricum F. Maekawa = Asarum heterotropoides F. Schmidt var. mandshuricum (Maxim.) Kitagawa*
#42	Saffron	*Crocus sativus Linne*
#43	Smilax Rhizome	*Smilax glabra Roxburgh*
#44	Gardenia Fruit	*Gardenia jasminoides Ellis*
#45	Notoginseng Root	*Panax notoginseng (Burkill) Feng Hwai Chen*
#46	Cornus Fruit	*Cornus officinalis Siebold et Zucc.*
#47	Zanthoxylum Fruit	*Zanthoxylum piperitum (L.) DC.*
#48	Jujube Seed	*Zizyphus jujuba Miller var. spinosa Hu ex H. F. Chou*
#49	Dioscorea Rhizome	*Dioscorea japonica Thunb.,*<br>*Dioscorea batatas Decne.*

#50	Sparganium Rhizome	*Sparganium stoloniferum Buchanan-Hamilton*
#51	Rehmannia Root	*Rehmannia glutiosa Libosch. var. purpurea Makino,*<br>*Rehmannia glutiosa Libosch. f. hueichingensis (Chao et Schih) Hsiao*
#52	Eleutherococcus Senticosus Rhizome	*Eleutherococcus senticosus Maximowicz*
#53	Chinese Wolfberry Root-bark	*Lycium chinense Miller*<br>*Lycium barbarum Linné*
#54	Tribulus Fruit	*Tribulus terrestris Linné*
#55	Paony Root	*Paeonia lactiflora Pallas*
#56	Plantago Seed,	*Plantago asiatica L.*
#57	Prepared Rehmannia Root	*Rehmannia glutinosa Liboschitz var. purpurea Makino*<br>*Rehmannia glutinosa Liboschitz*
#58	Amomum Seed	*Amomum xanthioides Wallich*
#59	Ginger	*Zingiber officinale Roscoe*
#60	Cimicifuga Rhizome	*Cimicifuga simplex Wormsk.*
#61	Magnolia Flower	*Magnolia salicifolia Maximowicz,*<br>*Magnolia biondii Pampanini,*<br>*Magnolia sprengeri Pampanini,*<br>*Magnolia heptapeta Dandy (Magnolia denudata Desrousseaux)*
#62	Gentiana Macrophylla Root	*Gentiana macrophylla Pallas,*<br>*Gentiana straminea Maximowicz,*<br>*Gentiana crassicaulis Duthie ex Burkill,*<br>*Gentiana dahurica Fischer*
#63	Red Paeony Root	*Paeonia lactiflora Pallas*
#64	Cnidium Rhizome	*Cnidium officinale Makino*
#65	Ginseng Root	*Panax ginseng C. A. Meyer (Panax schinseng Nees)*
#66	Atractylodes Lancea Rhizoma	*Atractylodes lancea De Candole*
#67	Mulberry Bark,<br>White Mulberry Root-bark	*Morus alba Linné*
#68	Mulberry Leaf	*Morus alba Linné,*<br>*Morus bombycis Koidzumi*
#69	Himalayan Teasel Root	*Dipsacus asperoides C.Y. Cheng et T.M. Ai*
#70	Perilla Herb	*Perilla frutescens (L.) Britton var. acuta Kudo*
#71	Rhubarb	*Rheum palmatum Linne*
#72	Jujube	*Zizyphus jujuba Miller var. inermis Rehder*
#73	Alisma Rhizome	*Alisma orientale Juzepczuk*
#74	Danshen Root	*Salvia miltiorhiza Bunge*
#75	Bamboo Culm	*Bambusa tuldoides Munro,*<br>*Phyllostachys nigra Munro var. henonis Stapf ex Rendle,*<br>*Phyllostachys bambusoides Siebold et Zuccarini*

#76	Panax Rhizome	*Panax japonicus C. A. Meyer*
#77	Anemarrhena Rhizome	*Anemarrhena asphodeloides Bunge*
#78	Dingxiang,<br>Caryophylli Flos	*Syzygium aromaticum Merrill et Perry (Eugenia caryophyllata Thunberg)*
#79	Uncaria Thorn	*Uncaria rhynchophylla (Miq.) Miq.*
#80	Polyporus Sclerotium	*Polyporus umbellatus Fries*
#81	Citrus Unshiu Peel	*Citrus unshiu Marcowicz,*<br>*Citrus reticulata Blanco*
#82	Gastrodia Tuber	*Gastrodia elata Blume*
#83	Asparagus Tuber	*Asparagus cochinchinensis Merrill*
#84	Benincasa Seed	*Benincasa cerifera Savi*<br>*Benincasa cerifera Savi forma emarginata K. Kimura et Sugiyama*
#85	Japanese Angelica root	*Angelica acutiloba Kitagawa,*<br>*Angelica acutiloba var. sugiyamae Hikino*
#86	Codonopsis Root	*Codonopsis pilosula (Franchet) Nannfeldt,*<br>*Codonopsis pilosula Nannfeldt var. modesta (Nannfeldt) L.T. Shen,*<br>*Codonopsis tangshen Oliver*
#87	Peach Kernel	*Prunus persica Batsch,*<br>*Prunus persica Batsch var. davidiana Maximowicz*
#88	Aralia Rhizome	*Aralia cordata Thunberg*
#89	Eucommia Bark	*Eucommia ulmoides Oliver*
#90	Desertliving Cistanche	*Cistanche deserticola Y.S.Ma,*<br>*Cistanche tubulosa (Schenk) R.Wight*
#91	Ginseng Root	*Panax ginseng C. A. Meyer (Panax schinseng Nees)*
#92	Fritillaria Bulb	*Fritillaria verticillata Willd. var. thunbergii Baker*
#93	Densefruit Pittany Root-bark	*Dictamnus dasycarpus Turczaninow*
#94	Ophiopogon Tuber	*Ophiopogon japonicus Ker-Gawler*
#95	Mentha Herb, Japanese Mint	*Mentha arvensis Linné var. piperascens Malinvaud*
#96	Pinellia Tuber	*Pinellia ternata Breitenb.*
#97	Angelica Dahurica Root	*Angelica dahurica (Fisch.) Benth. et Hook. fil.*
#98	Atractylodes Rhizome	*Atractylodes japonica Koidzumi ex Kitamura,*<br>*Atractylodes ovata DC.*
#99	Loquat Leaf	*Eriobotrya japonica Lindley*
#100	Areca	*Areca catechu Linné*
#101	Poria Sclerotium	*Poria cocos Wolf*
#102	Sinomenium Stem	*Sinomenium acutum Rehder et Wilson*
#103	Imperata Rhizome	*Imperata cylindrica Beauvois*
#104	Saposhnikovia Root	*Saposhnikovia divaricata Schischkin*
#105	Aconite,<br>Common Monkshood	*Aconitum carmichaeli,*<br>*Debeaux Aconitum japonicum Thunberg*

	Dauguter Root	
#106	Quercus Bark	*Quercus acutissima Carruthers,*<br>*Quercus serrata Murray,*<br>*Quercus mongolica Fischer ex Ledebour var. crispula Ohashi,*<br>*Quercus variabilis Blume*
#107	Malaytea Scurfpea Fruit	*Psoralea corylifolia Linné*
#108	Moutan Bark	*Paeonia suffruticosa Andrews*
#109	Ephedra Herb	*Ephedra intermedia Schrenk et C.A.May.,*<br>*Ephedra sinica Stapf,*<br>*Ephedra equisetina Bunge*
#110	Cannabis Fruit	*Cannabis sativa L.*
#111	Shrub Chaste Tree Fruit	*Vitex rotundifolia Linné filius*、<br>*Vitex trifolia Linné*
#112	Akebia Stem	*Akebia quinata Decne.,*<br>*Akebia trifoliata Koidz.*
#113	Myrrha	*Commiphora molmol Engler,*<br>*Balsamodendron ehrenbergianum Otto Karl (Carl) Berg*
#114	Bitter Cardamon	*Alpinia oxyphylla Miquel*
#115	Leonurus Herb	*Leonurus japonicus Houttuyn,*<br>*Leonurus sibiricus Linné*
#116	Coix Seed	*Coix lacryma-jobi L. var. ma-yuen Stapf*
#117	Japanese Gentian	*Gentiana scabra Bunge,*<br>*Gentiana manshurica Kitagawa,*<br>*Gentiana triflora Pallas*
#118	Alpinia Officinarum Rhizome	*Alpinia officinarum Hance*
#119	Ganoderma	*Ganoderma lucidum (Leysser ex Fries) Karsten, Ganoderma sinense Zhao, Xu et Zhang*
#120	Forsythia Fruit	*Forsythia suspensa Vahl,*<br>*Forsythia viridissima Lindley*

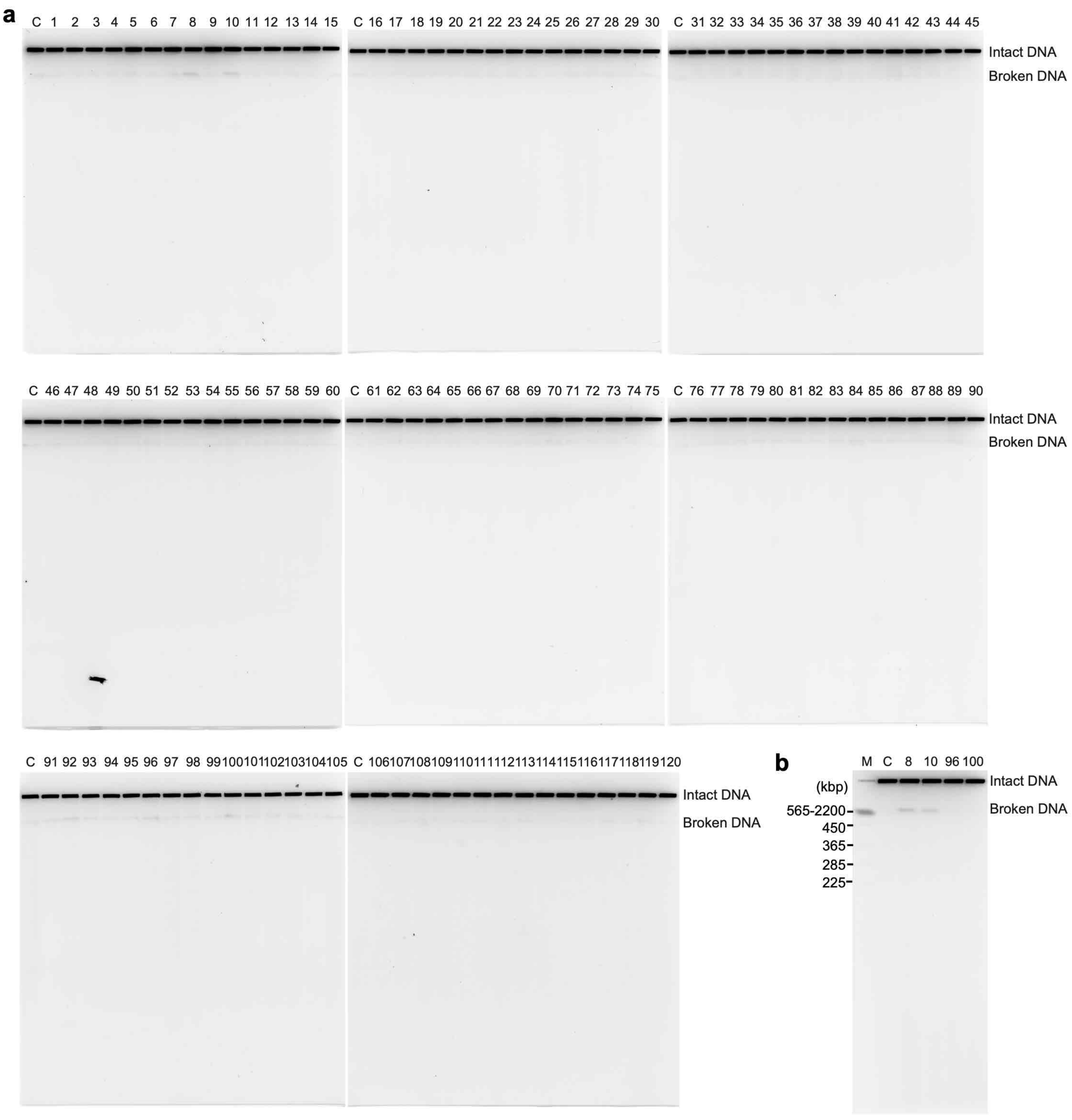

Supplementary Fig. 1. Screening of 120 herbal extracts by pulsed-field gel electrophoresis (PFGE). Cells were treated with each herbal extract at a concentration of 50 µg/mL for 24 h, and the accumulation of DSBs was analysed by PFGE. DNAs were visualized by ethidium bromide (EtBr) staining. **(a)** Results of primary screening. The number corresponds to the herbal medicine described in Table S1. **(b)** Results of secondary screening. M: DNA size marker (*S. cerevisiae* chromosomes). C: Untreated control.

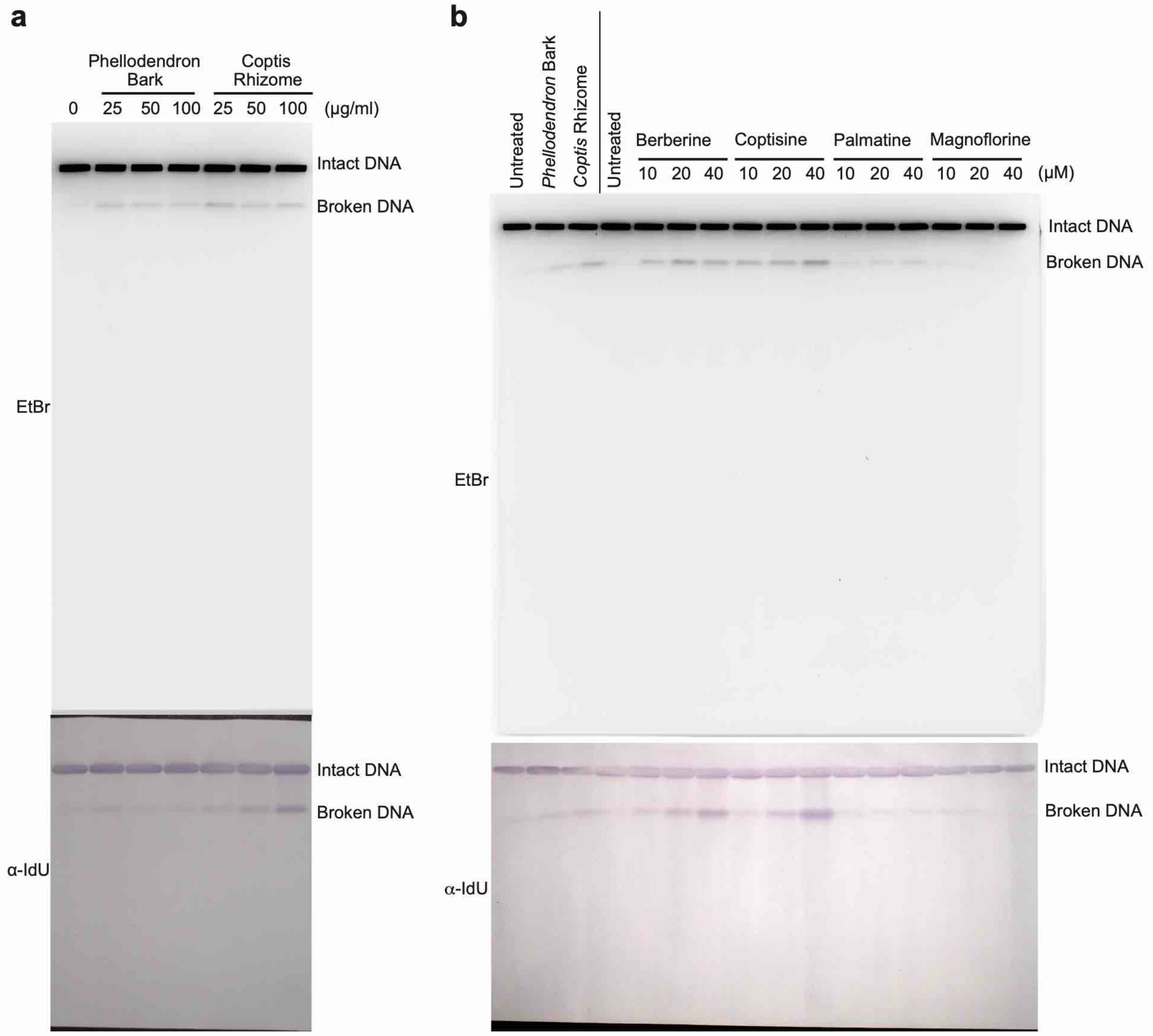

Supplementary Fig. 2. Complete gel images of the data shown in Figs. 1a and 1d. **(a)** Complete image of the EtBr-stained PFGE gel (upper) and the immunostained image (lower) presented in Fig. 1a. PFGE analysis of DSB accumulation after treatment with extracts of Phellodendron Bark and Coptis Rhizome. Total broken DNA was detected by ethidium bromide (EtBr) staining, whereas DSBs at DNA replication sites were detected by immunoblotting with anti-BrdU antibody. **(b)** Complete image of the EtBr-stained PFGE gel (upper) and the immunostained image (lower) presented in Fig. 1d. PFGE analysis of DSB accumulation after treatment with berberine, coptisine, palmatine, and magnoflorine.

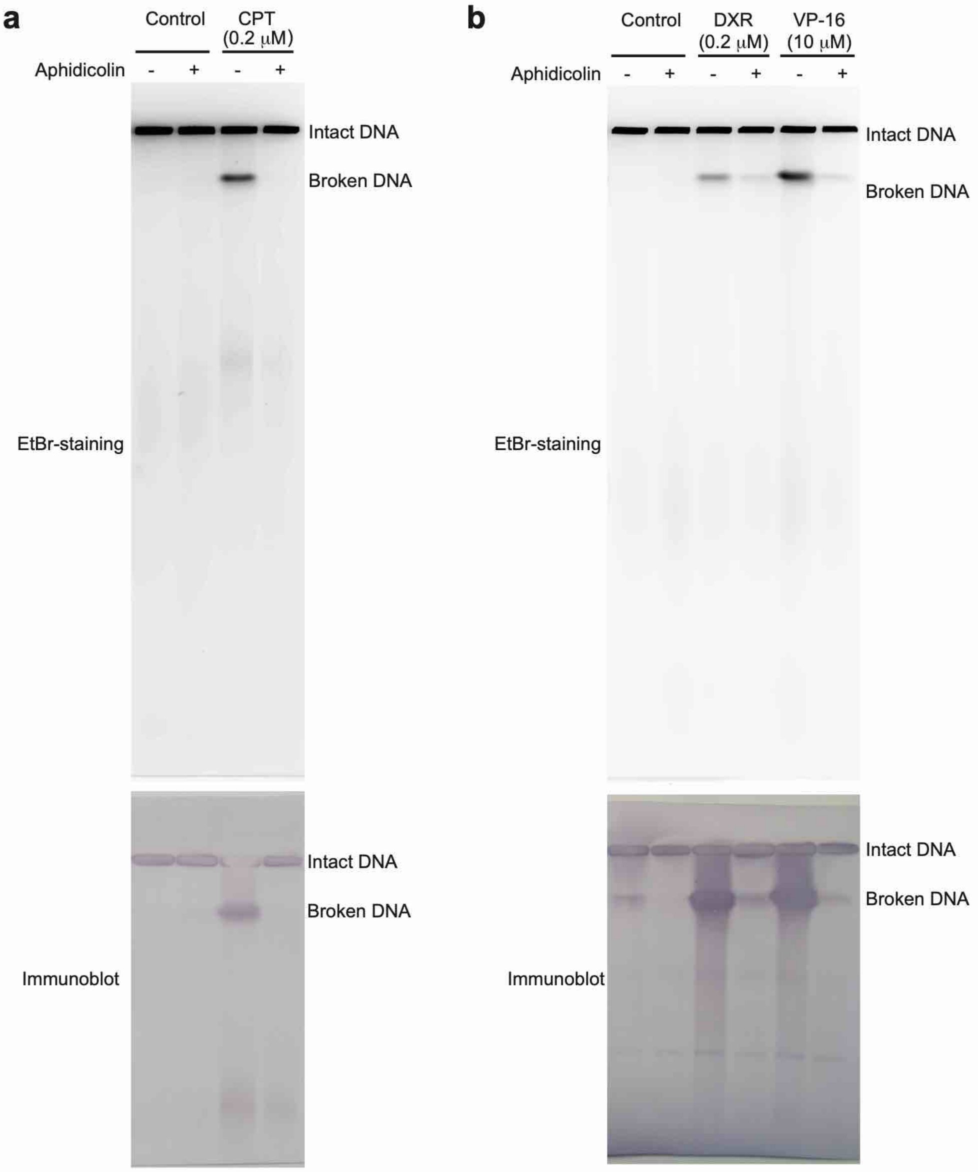

Supplementary Fig. 3. Suppression of DSBs after combined treatment with aphidicolin and topoisomerase poisoning. (a) PFGE analysis of DSB accumulation after treatment with aphidicolin combined with camptothecin (CPT). **(b)** PFGE analysis of DSB accumulation after treatment with aphidicolin combined with doxorubicin (DXR) and etoposide (VP-16).

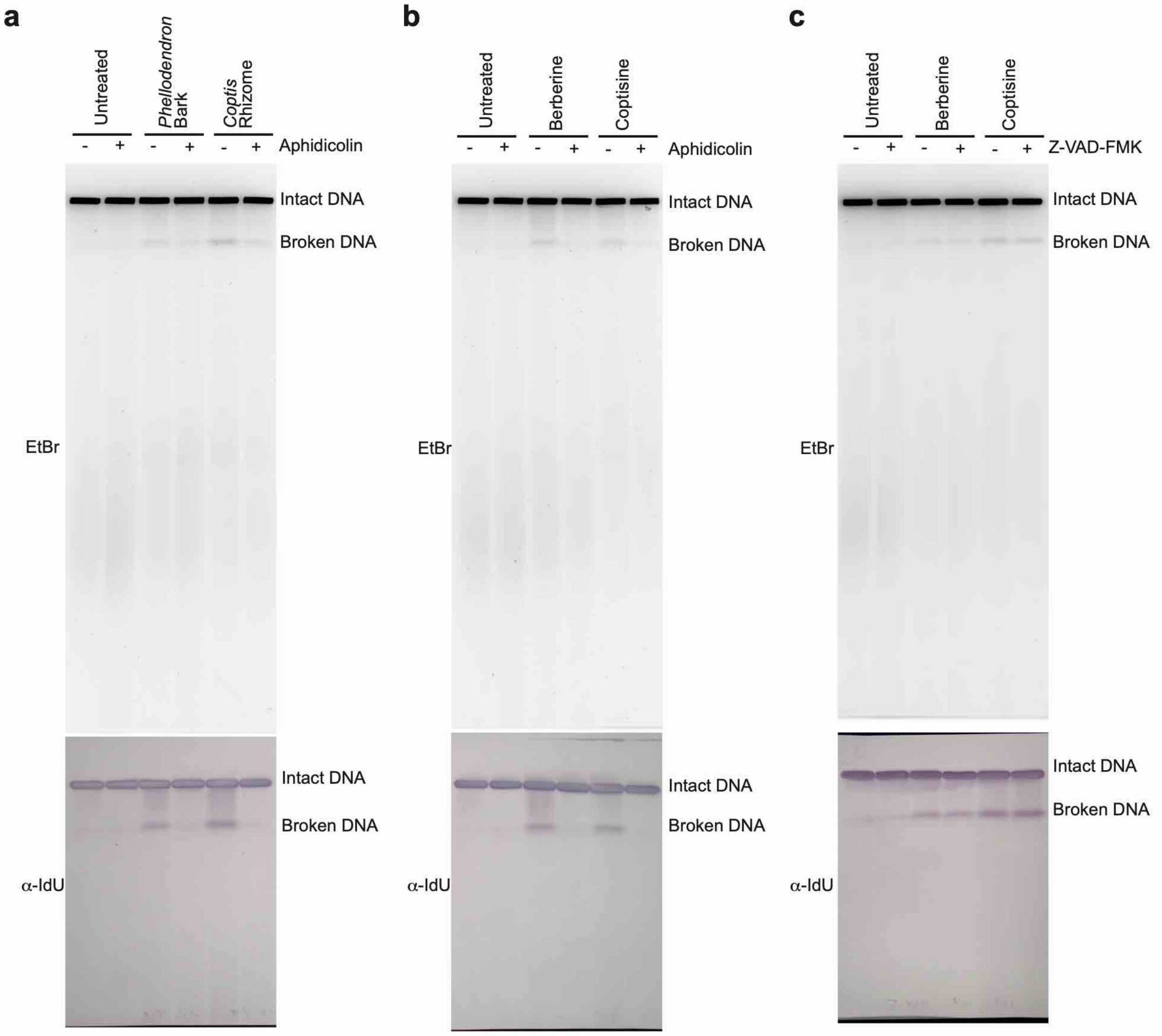

Supplementary Fig. 4. Complete gel images of the data shown in Figs. 2a-2c. **(a)** Complete image of the EtBr-stained PFGE gel (upper) and the immunostained image (lower) presented in Fig. 2a. PFGE analysis of DSB accumulation after treatment with aphidicolin combined with extracts of *Phellodendron Bark* and *Coptis Rhizome*. **(b)** Complete image of the EtBr-stained PFGE gel (upper) and the immunostained image (lower) presented in Fig. 2b. PFGE analysis of DSB accumulation after treatment with aphidicolin combined with extracts of berberine and coptisine. **(c)** Complete image of the EtBr-stained PFGE gel (upper) and the immunostained image (lower) presented in Fig. 2c. PFGE analysis of DSB accumulation after treatment with Z-VAD-FMK combined with extracts of berberine and coptisine.

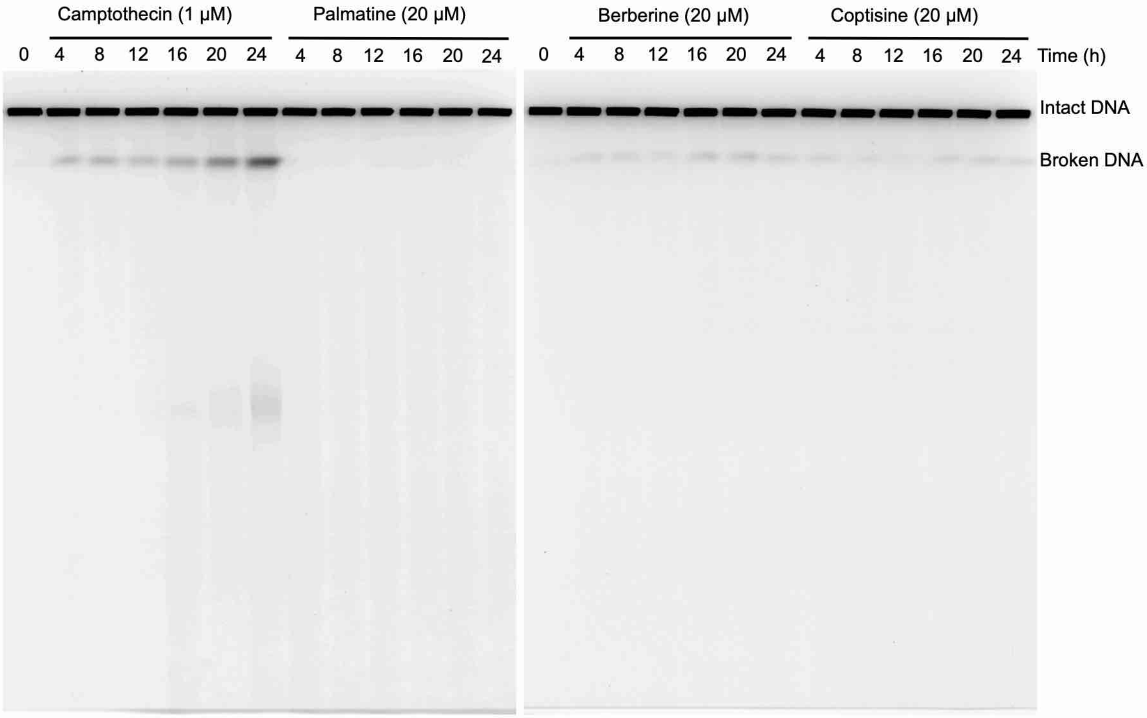

Supplementary Fig. 5. Complete gel images of the data shown in Fig. 2d. Complete image of the EtBr-stained PFGE gel. Time-course PFGE analysis of DSB accumulation after treatment with CPT, palmatine, berberine and coptisine.

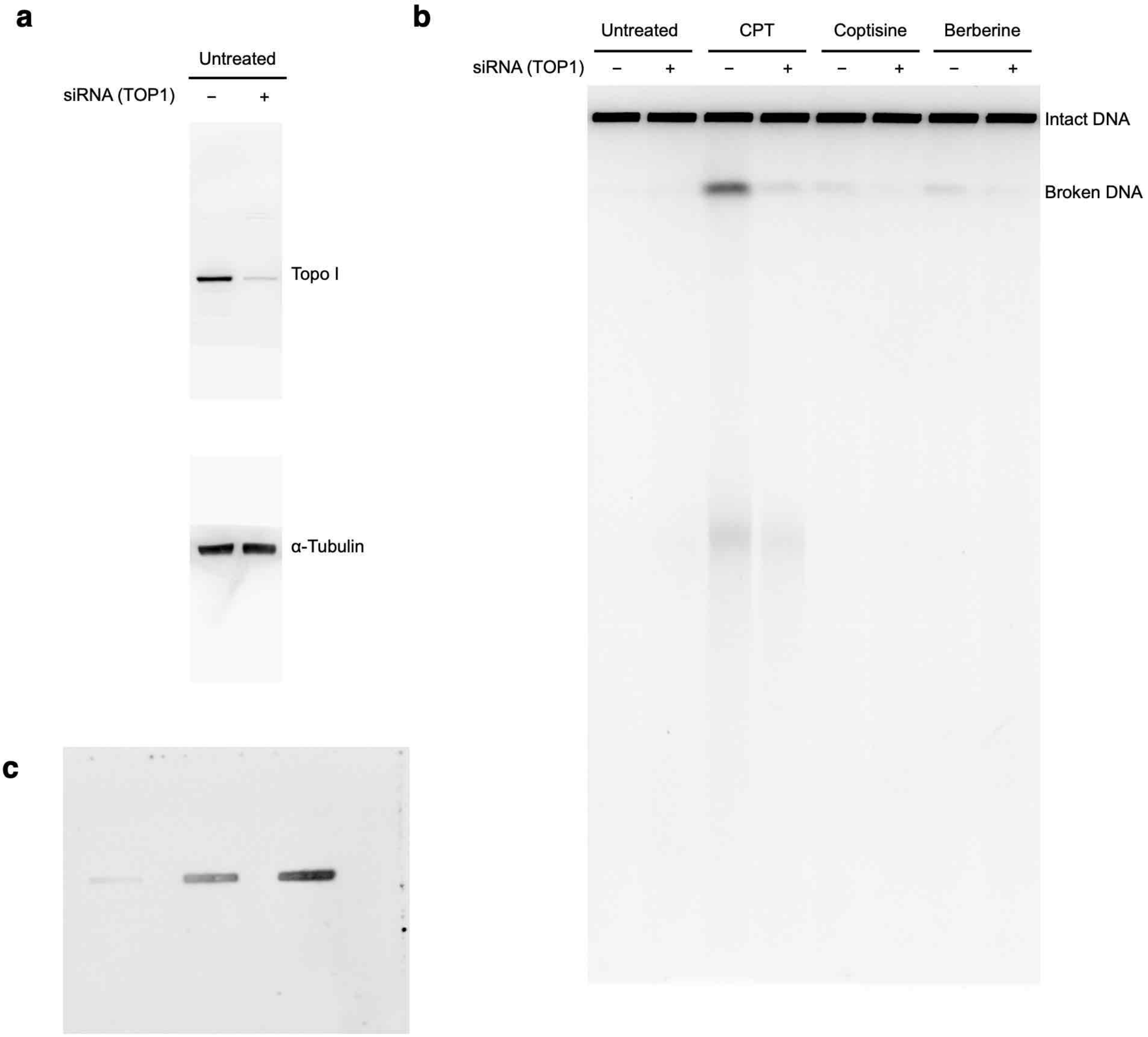

Supplementary Fig. 6. Complete gel images of the data shown in Figs. 3a,3b, and 3d. (a) Complete gel images of the Western blot analysis of Topo I and α-tubulin (Fig. 3a). α-Tubulin was used as a loading control. **(b)** Complete gel image of the PFGE analysis of DSB formation after treatment with CPT, coptisine and berberine in the absence of Topo I presented in Fig. 3b. **(c)** The original image of ICE assay blots.

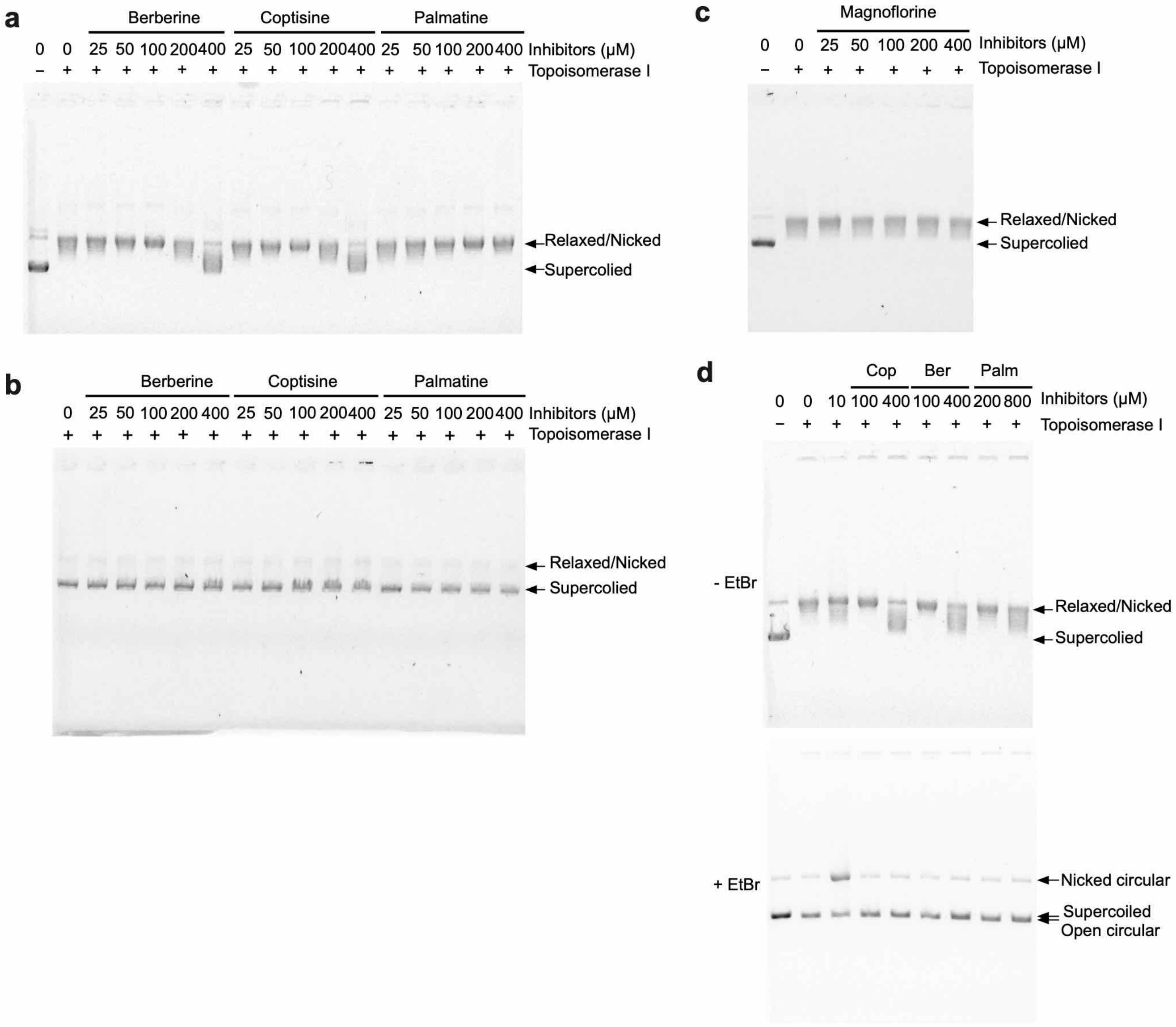

Supplementary Fig. 7. Complete gel images of the data shown in Figs. 4a-4d. (a, b) Complete gel images of the relaxation activity of Topo I presented in Figs. 4a and 4b. Inhibition of the relaxation activity of Topo I by berberine, coptisine, and palmatine. **(c)** Complete gel images of the relaxation activity of Topo I presented in Fig. 4c. Complete gel images of the relaxation activity of Topo I. Relaxation activity of Topo I after treatment with magnoflorine. **(d)** Complete gel images of the relaxation activity of Topo I presented in Fig. 4d. Detection of nicked DNA due to the relaxation activity of Topo I after treatment with CPT, coptisine (Cop), berberine (Ber), and palmatine (Palm).

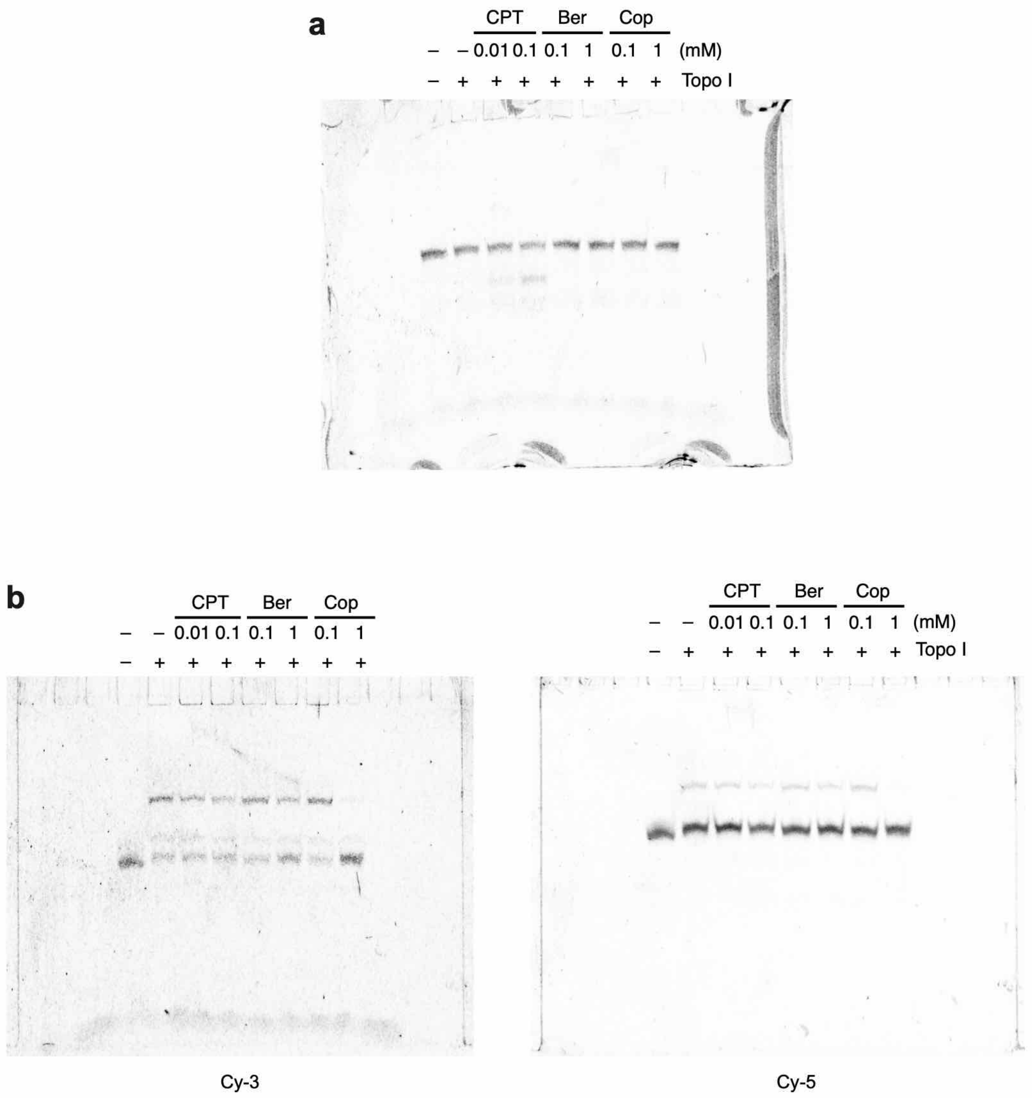

Supplementary Fig. 8. Complete gel images of the data shown in Figs. 5b and 5d. (a) Complete gel images of the nicking activity of Topo I presented in Figs. 5b. Inhibition of the relaxation activity of Topo I by berberine, coptisine, and palmatine. **(b)** Complete gel images of the rejoining activity of Topo I presented in Fig. 5d. Detection of nicking and rejoining activity of Topo I after treatment with CPT, coptisine (Cop), berberine (Ber), and palmatine (Palm). (e) Complete gel image of nicking assay

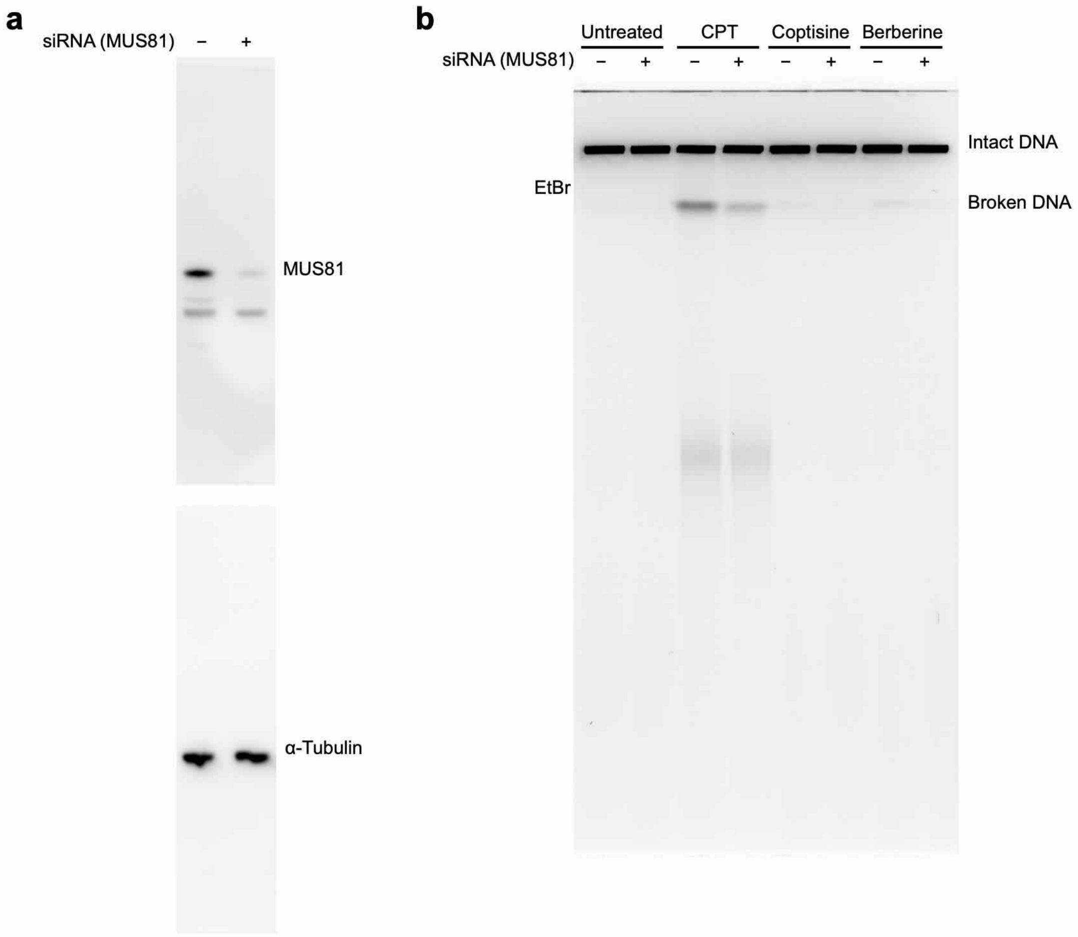

Supplementary Fig. 9. Complete gel images of the data shown in Figs. 6a and 6b. (a) Complete gel images of the Western blot analysis of mMUS81and α-tubulin (Fig. 6a). α-Tubulin was used as a loading control. **(b)** Complete gel image of the PFGE analysis of DSB formation after treatment with CPT, coptisine and berberine in the absence of MUS81 presented in Fig. 3b.

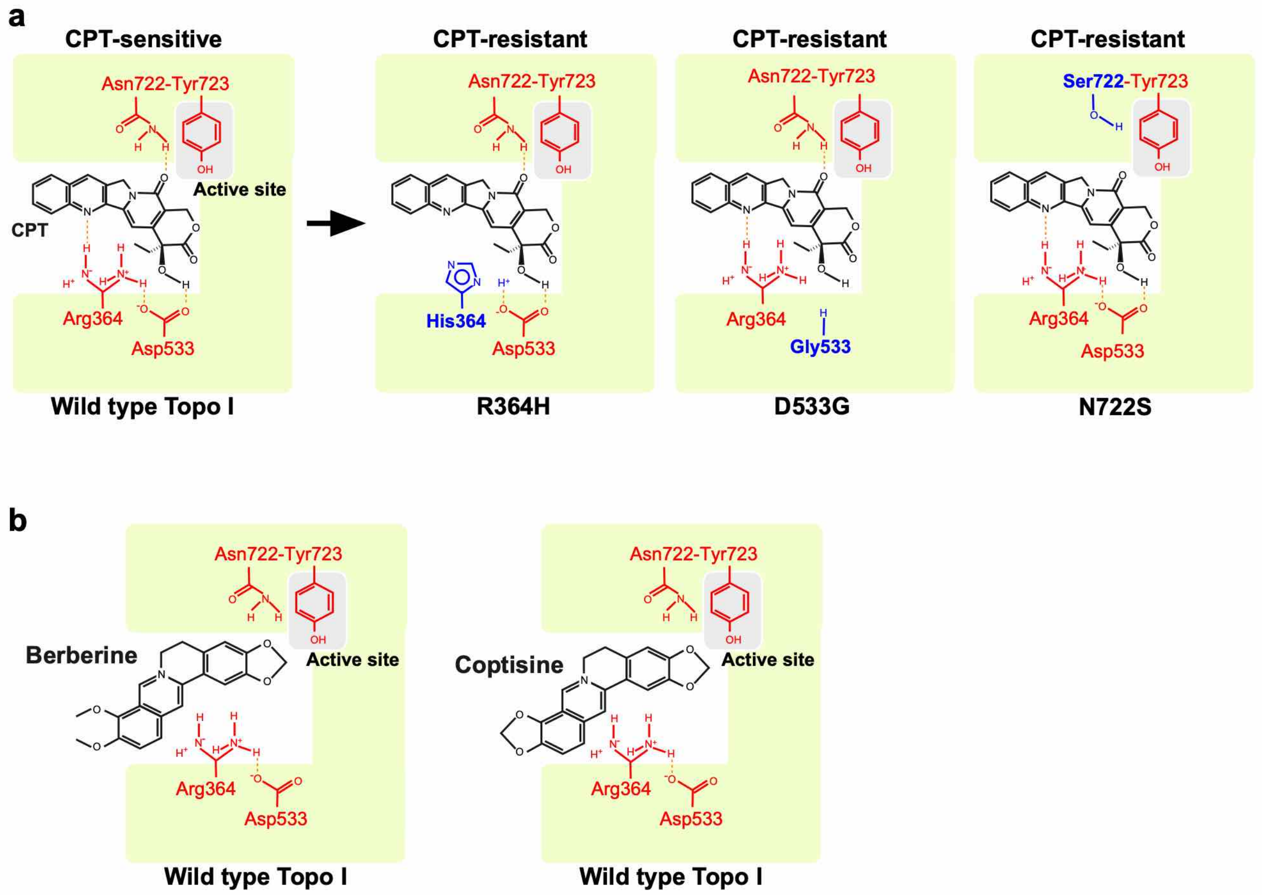

Supplementary Fig. 10. Hypothesized binding modes of Topo I with berberine and coptisine. (a) Interaction domains of CPT with the active site of Topo I. The 364th Arg, 533rd Asp and 722nd Asn residues of Topo I are responsible for the interaction with CPT. Defective interaction of CPT with the active site of CPT-resistant Topo I. **(b)** Conceptualization of the mechanism of action of berberine and coptisine that enables their activity against CPT-resistant Topo I. These BIAs act against CPT-resistant mutants because they do not possess the functional groups responsible for interactions with Topo I.

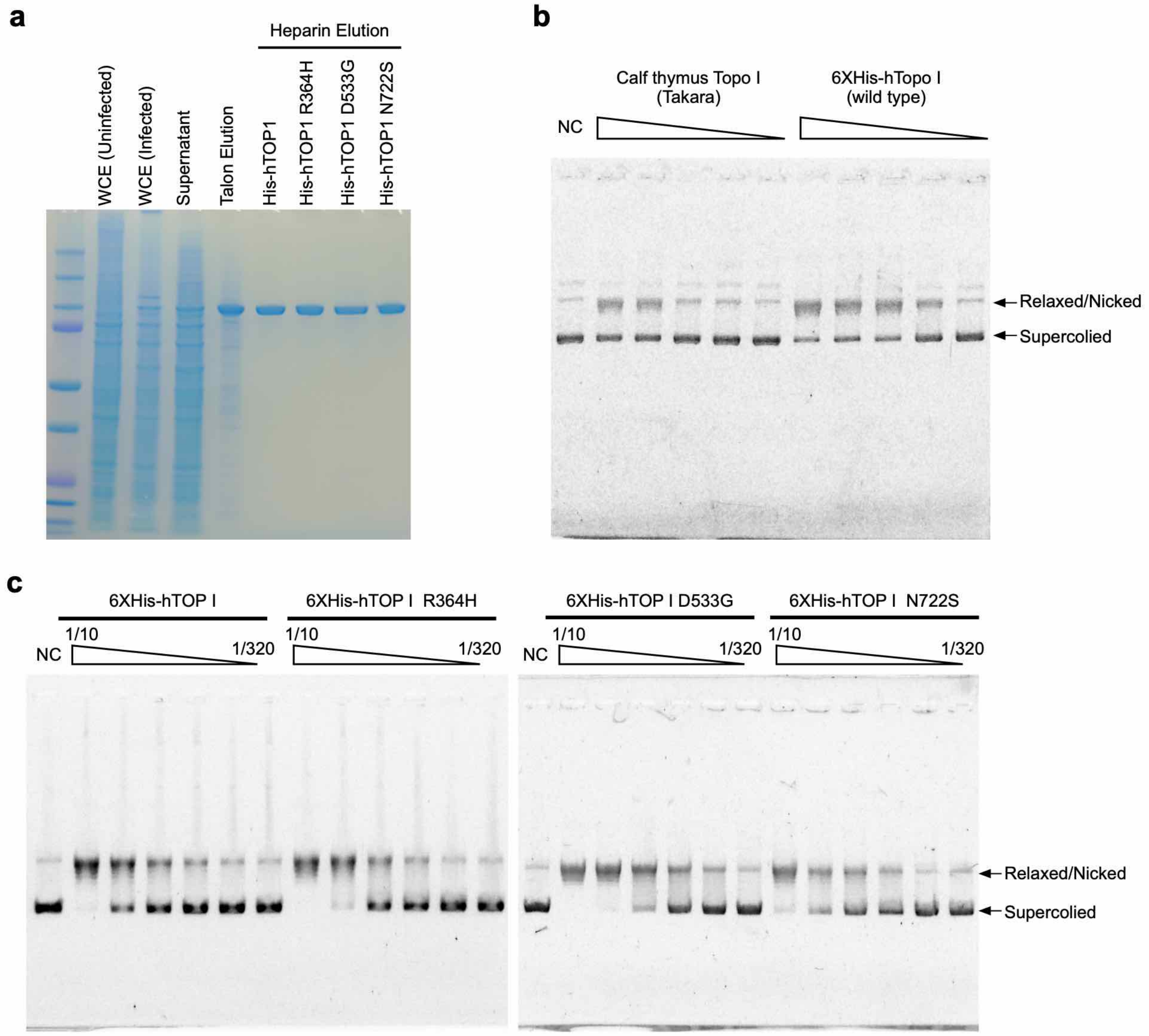

Supplementary Fig. 11. Purification of CPT-resistant Topo I. (a) SDS-PAGE of purified wild-type and CPT-resistant Topo I proteins, such as R364H, D533G or N722S mutants. **(b)** Comparison of the activities between calf thymus Topo I purchased from Takara and his-tagged Topo I purified in this study. **(c)** Relaxation activities of various Topo I-mutated proteins.

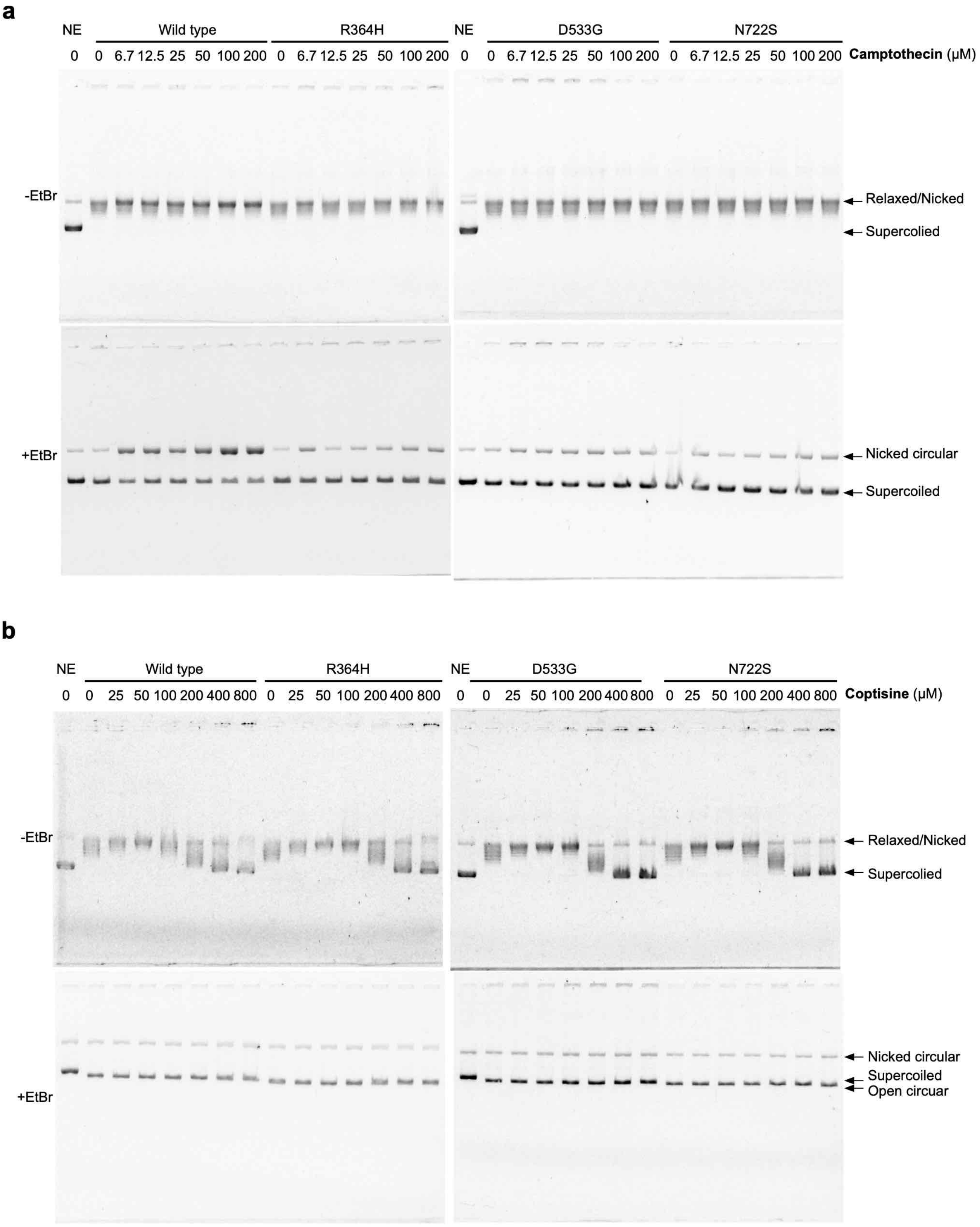

Supplementary Fig. 12. Complete gel images of the data shown in Figs. 7b and 7c. **(a)** Complete gel images of the relaxation activity of Topo I presented in Fig. 7b. Relaxation activity of CPT-resistant Topo I proteins in the presence of CPT in vitro. **(b)** Complete gel images of the relaxation activity of Topo I presented in Fig. 7c. Inhibitory effect of coptisine on CPT-resistant Topo I proteins in vitro.

c

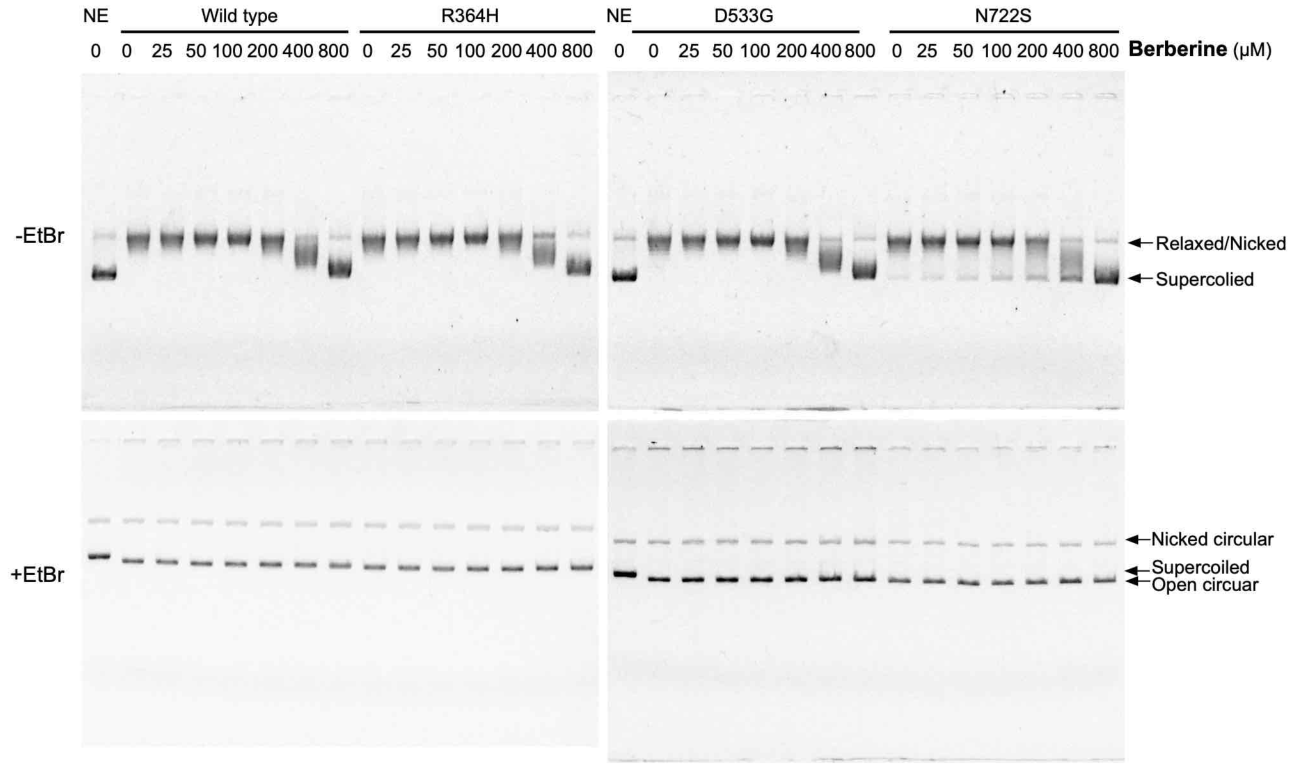

d

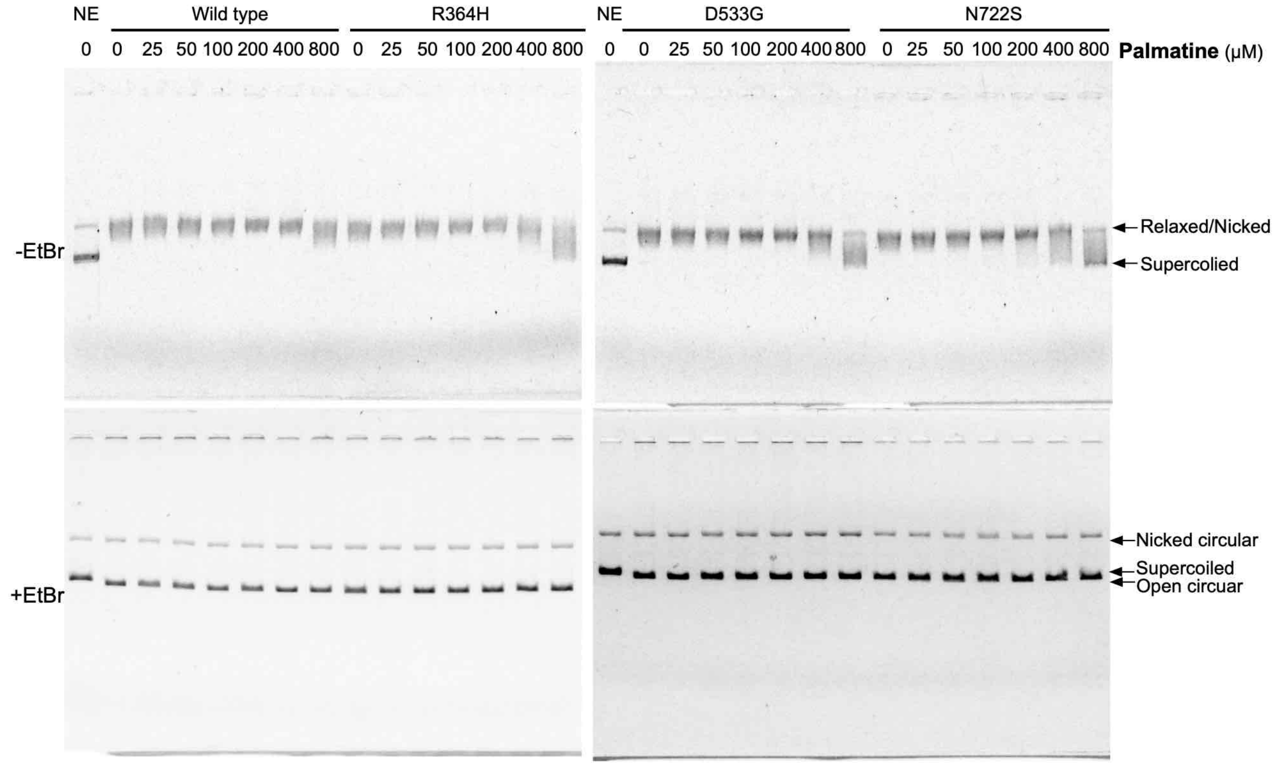

Supplementary Fig. 12 (Continued). Complete gel images of the data shown in Figs. 7d and 7e. (c, d) Complete gel images of the relaxation activity of Topo I presented in Figs. 7d and 7e. Inhibitory effect of berberine **(c)** and palmatine **(d)** on CPT-resistant Topo I proteins in vitro.

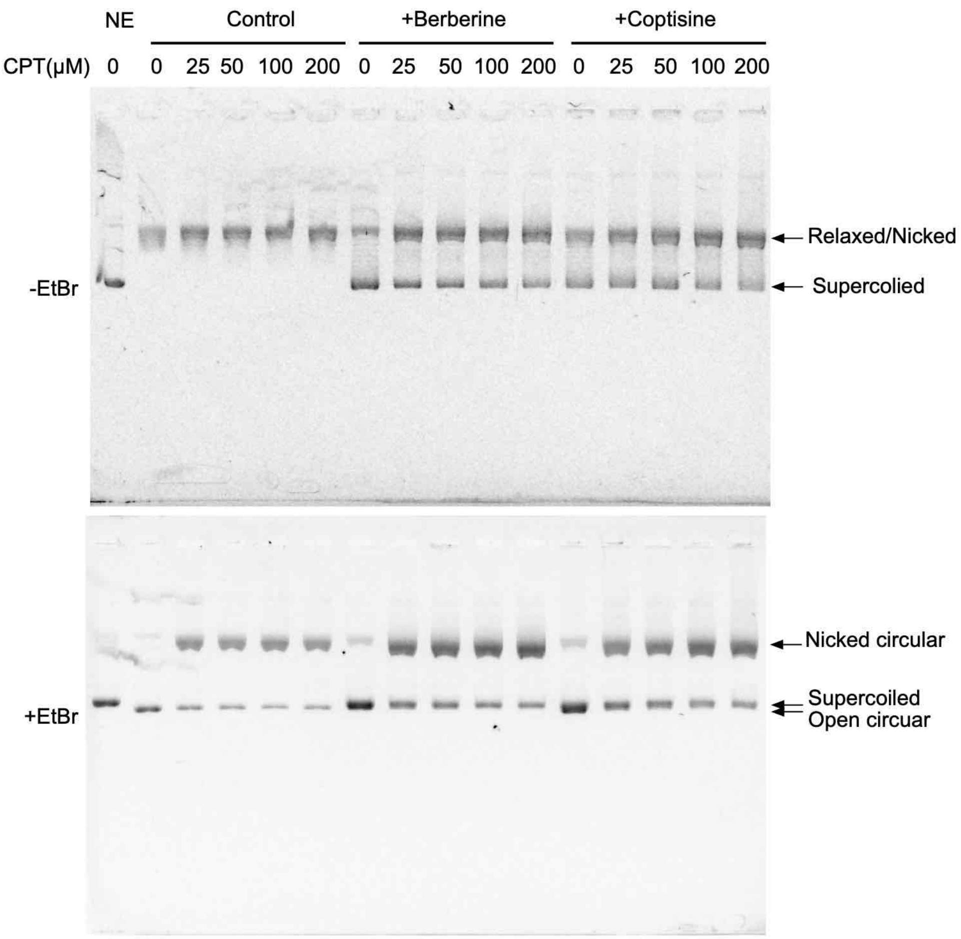

Supplementary Fig. 13. Complete gel images of the data shown in Fig. 9a. Complete gel images of the relaxation activity of Topo I presented in Fig. 9a. Relaxation activity of CPT-resistant Topo I proteins in the presence of CPT in vitro.

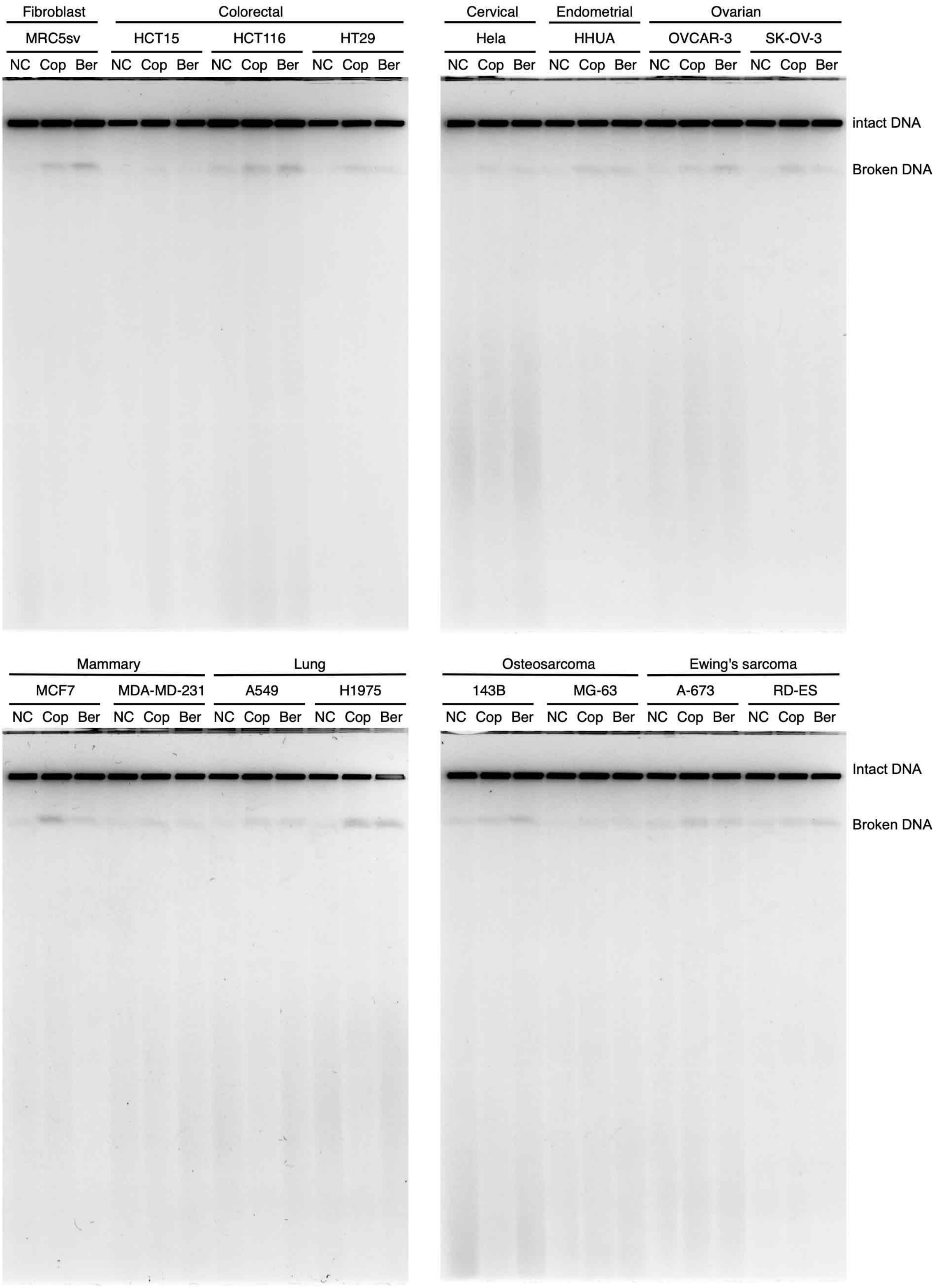

Supplementary Fig. 14. Complete gel images of the data shown Fig. 10. Complete image of the EtBr-stained PFGE gel. The cells were incubated with 10 μM coptisine (Cop) and berberine (Ber) for 24 h, and the accumulation of DSBs was analysed by PFGE.
